# AIE-doped poly(ionic liquid) photonic spheres: a single sphere-based customizable sensing platform for the discrimination of multi-analytes†
†Electronic supplementary information (ESI) available: Synthesis and characterization of the AIE luminogen, experimental details, response profiles and results of the multivariate analysis. See DOI: 10.1039/c7sc02409f



**DOI:** 10.1039/c7sc02409f

**Published:** 2017-06-30

**Authors:** Wanlin Zhang, Ning Gao, Jiecheng Cui, Chen Wang, Shiqiang Wang, Guanxin Zhang, Xiaobiao Dong, Deqing Zhang, Guangtao Li

**Affiliations:** a Department of Chemistry , Key Lab of Organic Optoelectronics and Molecular Engineering , Tsinghua University , Beijing 100084 , P. R. China . Email: lgt@mail.tsinghua.edu.cn; b Institute of Chemistry , Chinese Academy of Sciences , Beijing 100190 , P. R. China . Email: dqzhang@iccas.ac.cn

## Abstract

By simultaneously exploiting the unique properties of ionic liquids and aggregation-induced emission (AIE) luminogens, as well as photonic structures, a novel customizable sensing system for multi-analytes was developed based on a single AIE-doped poly(ionic liquid) photonic sphere. It was found that due to the extraordinary multiple intermolecular interactions involved in the ionic liquid units, one single sphere could differentially interact with broader classes of analytes, thus generating response patterns with remarkable diversity. Moreover, the optical properties of both the AIE luminogen and photonic structure integrated in the poly(ionic liquid) sphere provide multidimensional signal channels for transducing the involved recognition process in a complementary manner and the acquisition of abundant and sufficient sensing information could be easily achieved on only one sphere sensor element. More importantly, the sensing performance of our poly(ionic liquid) photonic sphere is designable and customizable through a simple ion-exchange reaction and target-oriented multi-analyte sensing can be conveniently realized using a selective receptor species, such as counterions, showing great flexibility and extendibility. The power of our single sphere-based customizable sensing system was exemplified by the successful on-demand detection and discrimination of four multi-analyte challenge systems: all 20 natural amino acids, nine important phosphate derivatives, ten metal ions and three pairs of enantiomers. To further demonstrate the potential of our spheres for real-life application, 20 amino acids in human urine and their 26 unprecedented complex mixtures were also discriminated between by the single sphere-based array.

## Abstract 2

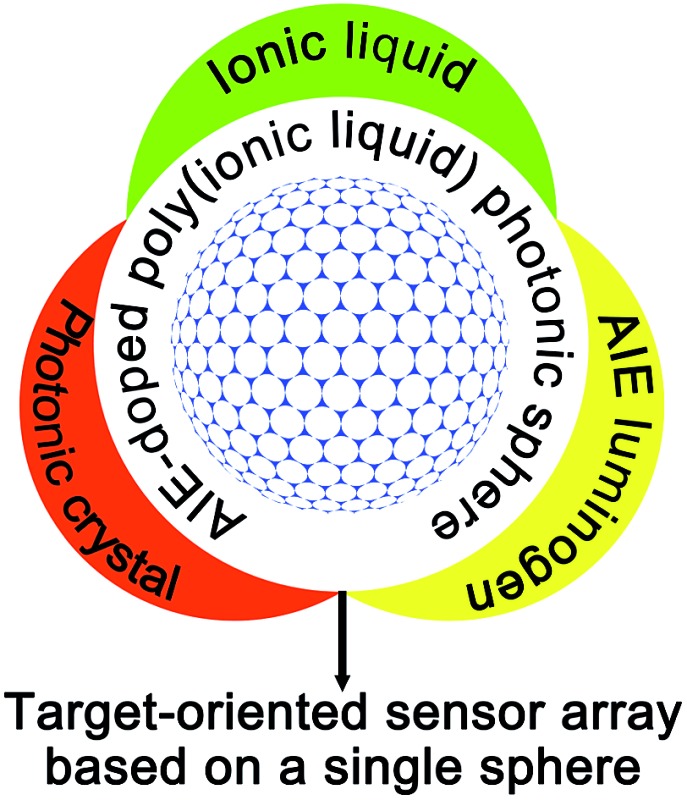
Based on one single AIE-doped photonic-structured polyionic liquid sphere, a novel customizable virtual sensor array system was developed.

## Introduction

Differential sensing has emerged as an effective and powerful tool for multi-analyte detection and discrimination, especially in complex mixtures. In comparison to traditional “lock-and-key” based molecular sensing, where a highly specific receptor with strong affinity is required, the approach of differential sensing relies on fingerprint information collected from the interaction of an individual analyte or complex mixtures with an array of cross-reactive receptors and their resulting characteristic response patterns.^1^ Obviously, for the successful implementation of differential sensing, the “ideal” strategy to develop a desired sensing array should possess the following favorable features. Firstly, a sufficient number of receptors or sensing elements with different selectivities should be rapidly and conveniently generated through the devised method. Secondly, the collected sensing elements should exhibit high diversity after interacting with multi-analytes, hence allowing more information to be extracted for the discrimination and identification of the target analytes. Ideally, a single element should provide sufficient sensing information and serve as a virtual sensor array to realize the miniaturization of a constructed sensing device.^2^ Thirdly, as an attractive feature, the universal applicability or broad utility of the created array is also highly desirable. Thus, the array is expected to be a sensing platform with high flexibility and extendibility that can be facilely tailored and customized for the discrimination of a broad spectrum of multi-analytes. Finally, the used sensing protocol should be handy. Over the past few years, numerous strategies for creating sensor arrays have been developed and successfully employed for the detection and discrimination of various important analytes.^3–9^ Although great progress has been made in differential sensing, the reported strategies with all of the desired features mentioned above are still limited.

**Fig. 1:**
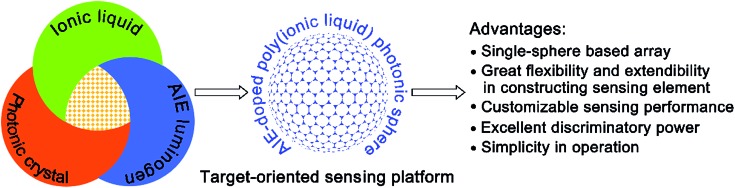
General illustration of the construction of an AIE-doped poly(ionic liquid) photonic sphere as a target-oriented sensing platform for multi-analytes and the prominent advantages of this platform.

In this article, the microfluidic synthesis of poly(ionic liquid) (PIL) inverse opal spheres doped with an aggregation-induced emission (AIE) dye is described. Interestingly, it was found that by exploiting the unique properties of ionic liquids (ILs),^10^ photonic structures,^11^ and AIE luminogens,^12^ the fabricated sphere could be used as a single sphere-based customizable platform with distinct advantages for the target-oriented differential sensing of a broad spectrum of multi-analytes (Fig. 1). Such a strategy allows for the facile formation of a large number of sphere-based sensing elements through a simple counterion exchange reaction. Due to the nearly unlimited combinations of cations and anions in ILs, a huge pool or library of sensing elements could be conveniently and rapidly established from the parent PIL photonic sphere. Importantly, we found that the extraordinary multiple types of molecular interactions involved in ILs, including van der Waals forces, electrostatic forces, hydrogen bonding, hydrophobic interactions, and π–π interactions,^13^ provide tremendous binding diversity upon interaction with multi-analytes, affording distinct response patterns for discrimination. Moreover, as competent multi-channel signal transducers, the inherent optical properties of both the photonic structure of the used sphere and the integrated AIE luminogen are sensitive to environmental changes, and the obtained complementary signals from fundamentally different transduction principles provide more efficient information,^14^ enabling enhanced discriminatory performance with a miniaturized array. In fact, in our case, one AIE-doped PIL photonic sphere alone can generate abundant sensing information, which is enough for precisely discriminating complex systems, and namely a “lab-on-a-sphere” concept is achieved. What is more appealing, based on the “task-specific” concept of ILs,^15^ is that we also found that different selective receptor species as counterions could be facilely incorporated into the prepared photonic spheres by anion exchange, and thus the recognition performance of the spheres could be designed to be biased towards particular analyte classes of interest. This attribute indicates that the photonic sphere described here could serve as a single sphere-based customizable sensing platform with high flexibility and extendibility for the on-demand detection and discrimination of a broad spectrum of analytes. Finally, the microfluidic synthetic approach guarantees the reliable and reproducible preparation of uniform photonic spheres on a multi-gram scale, and the sphere-based sensing protocol is more convenient compared to solution-based sensing processes. As a demonstration of the excellent discriminatory power of our new strategy, in this work all 20 natural amino acids and their 26 complex mixture systems, nine important phosphate derivatives, ten metal ions and three pairs of dicarboxylic acid enantiomers were successfully detected and discriminated between on a single AIE-doped PIL photonic sphere with 100% accuracy.

## Results and discussion

### Preparation of the AIE-doped poly(ionic liquid) photonic sphere

**Fig. 2:**
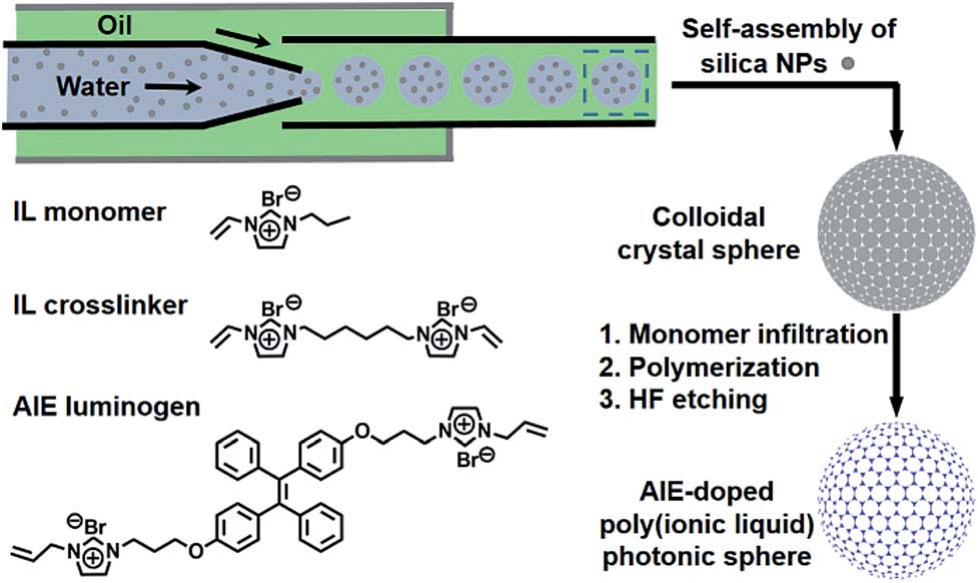
Schematic illustration of the preparation of the AIE-doped poly(ionic liquid) photonic sphere and the chemical structures of the ionic liquid monomer, crosslinker and AIE luminogen used.

**Fig. 3:**
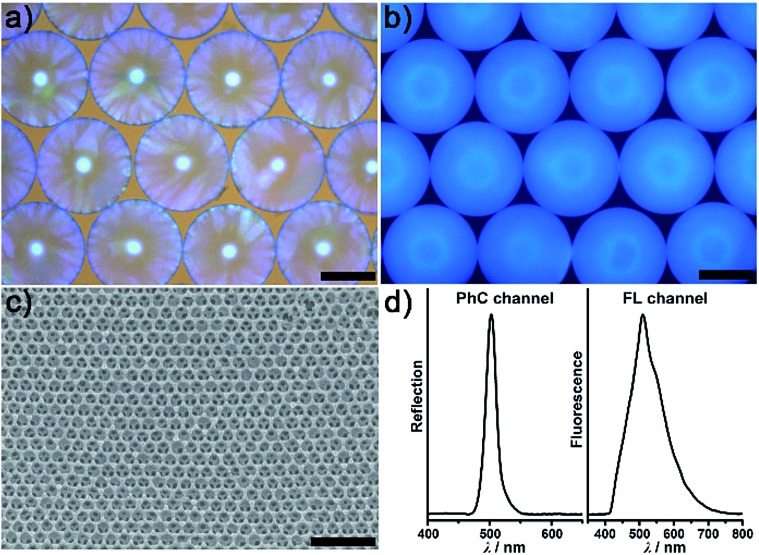
(a) Optical image of the parent AIE-doped poly(ionic liquid) photonic spheres. (b) Fluorescence image of the parent AIE-doped poly(ionic liquid) photonic spheres (false color, exposure time 200 ms). (c) SEM image of the parent AIE-doped poly(ionic liquid) photonic spheres with an ordered macropore structure. (d) Reflection (left) and fluorescence (right) spectra of the parent AIE-doped poly(ionic liquid) photonic spheres. Scale bars in (a), (b) and (c) are 200 μm, 200 μm and 500 nm, respectively.

Fig. 2 illustrates the general process for the preparation of the AIE-doped poly(ionic liquid) inverse opal photonic spheres, as well as the structures of the imidazolium-based IL monomer, crosslinker, and tetraphenylethylene-type AIE luminogen used in this work for the formation of the spheres. Fig. S1 and S2 in the ESI† show their synthetic routes and the corresponding characterizations. The monodisperse SiO_2_ colloidal crystal spheres were firstly prepared using a microfluidic approach and were then employed as photonic templates. After the infiltration of monomer solution into the interstices of the templates, the subsequent polymerization followed by the removal of the SiO_2_ nanoparticles using HF afforded the AIE-doped inverse opal PIL spheres. In our case, uniform SiO_2_ nanoparticles with a diameter of 170 nm were used to microfluidically generate the spherical template (Fig. S3†), and the monomer solution was composed of the synthesized IL monomer, AIE luminogen, and crosslinker, as well as the photoinitiator, for the performed polymerization. Fig. 3a and c display the optical and SEM images of the resultant AIE-doped PIL spheres with 3D-ordered macropore structures. Owing to the shrinkage of the poly(ionic liquid) hydrogel in the dry state, the observed pore diameter in Fig. 3c is smaller than that of the used silica nanoparticles. In fact, a similar phenomenon is often observed in the literature.^11*l*^ The ordered macropore structure endows the PIL spheres with photonic properties (Fig. 3a). At the same time, the doped AIE luminogen imparts the PIL spheres with strong fluorescence (Fig. 3b). This unique photonic crystal (PhC) optical property, together with the fluorescence (FL), provides two complementary transducer channels (Fig. 3d) which can directly and sensitively report the binding events of PIL with analytes. More importantly, as a sensing element, the PIL-based sphere can easily be converted into a huge number of sphere arrays through a simple counterion exchange reaction and compared to the covalent synthetic approaches described in the literature,^1^ this noncovalent method should be a more convenient way to facilely access various sensing elements from one parent PIL sphere.^10,16^ As a key point, the unique multiple intermolecular interaction feature of the IL units imparts the PIL spheres with high cross-reactivity upon interaction with different analytes.^17^ Thus, with rich response information obtained, a single PIL sphere could serve as a virtual sensor array for a wide range of multi-analytes.

### Single sphere-based array for 20 natural amino acids

**Fig. 4:**
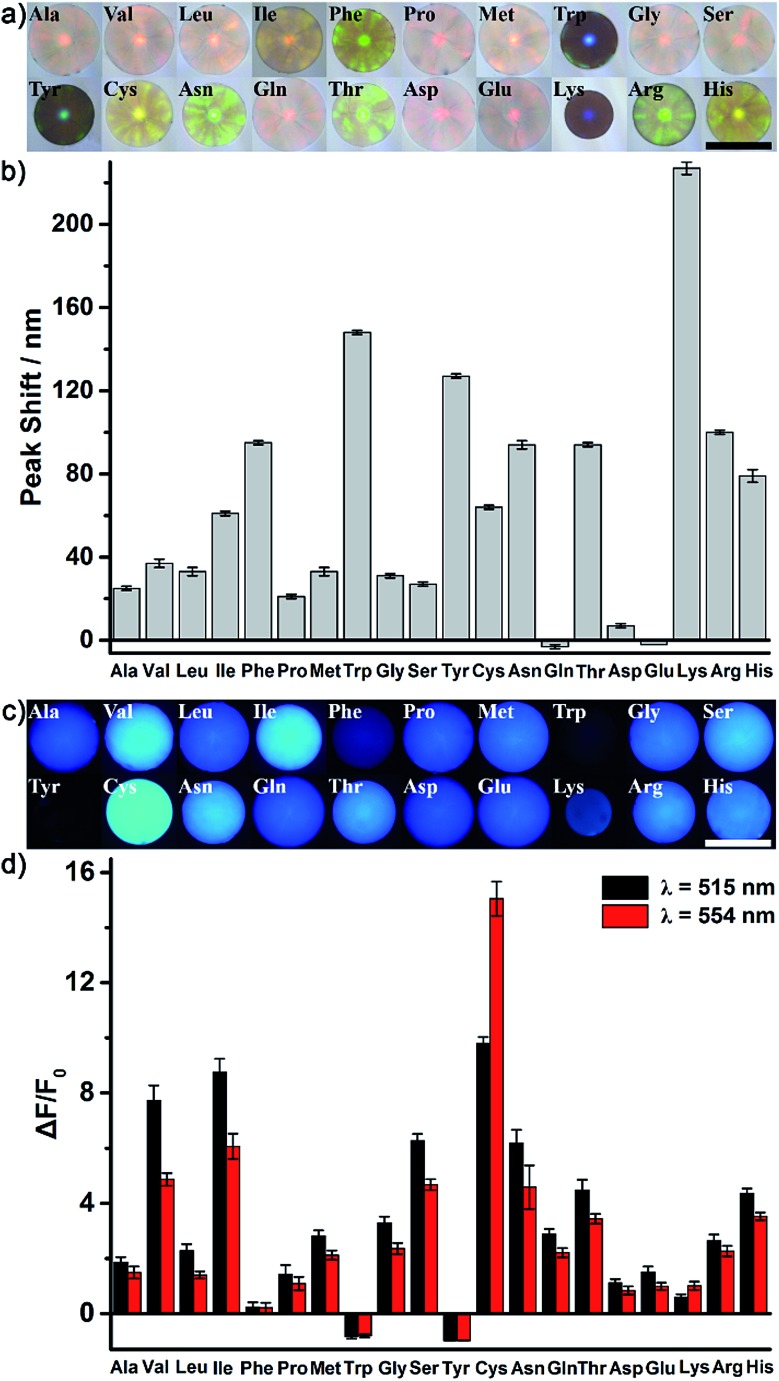
The responses of the AIE-doped poly(ionic liquid) photonic spheres (OH^–^ form) to the 20 natural amino acids at 10 mM. (a) Optical images. (b) Histogram of reflection peak shifts. (c) Fluorescence images (false color, exposure time 30 ms). (d) Histogram of the folds of fluorescence enhancement. Scale bars in (a) and (c) are 500 μm.

As a proof of our concept, the identification of all 20 natural amino acids (Fig. S4†) was first chosen to demonstrate the excellent discrimination power of the AIE-doped PIL photonic sphere. In this case, the PIL photonic spheres of the OH^–^ form (Fig. S5†) were simply incubated in the analyte solutions for the discrimination task. After nonspecifically binding the amino acids with the PIL spheres, the response signals from the PhC and FL channels were gathered and are shown in Fig. 4 and S6.† Fig. 4a and b show the optical images and the corresponding PhC responses of the PIL spheres upon exposure to the analytes. Expectedly, due to the unique multiple intermolecular interactions involved in the imidazolium-based ionic liquids, including electrostatic forces, hydrogen bonding, hydrophobic interactions, π–π interactions and van der Waals forces, the response of one PIL sphere to all of the amino acids alone was already very sensitive and complicated. Depending on the individual structural features of the amino acids, their interactions with the PIL spheres induced the shrinking or swelling of the PIL photonic spheres to a varying extent, thus generating obvious and diverse wavelength shifts of Bragg diffraction (Fig. 4b). Even a subtle difference, for example between Asn and Gln, could cause different responses which were clearly detectable on the PhC channel. Accompanying the diffraction shifts, distinct color changes were also observed (Fig. 4a) even by the naked eye when the PIL photonic spheres were exposed to Phe, Trp, Tyr, Asn, Thr, *etc.* Interestingly, Lys is a typical basic amino acid with two amino groups and one carboxyl group and has the largest shift of the Bragg diffraction peak (227 nm) compared to all of the other amino acids. A reasonable explanation for this observation is that the acidic hydrogens at the C2 position on the imidazolium rings are hydrogen bond donors which exhibit a high binding affinity with amino groups.^18^

Besides the photonic responses, after the interaction with different amino acids, the fluorescence behavior of the AIE luminogen doped in the PIL photonic spheres also simultaneously varied in a “turn on” fashion with the shrinking or swelling of the PIL spheres, as well as with the change in their micro-environments, as shown in Fig. 4c and d. We monitored the variation of the fluorescence intensity at 515 nm and 554 nm (*F*_515 nm_ for peak, *F*_554 nm_ for shoulder), which dramatically increased in most of the amino acids on light-up mode. For example, compared to the initial intensity of the fluorescence of the PIL sphere at 515 nm, *ca.* 7.7-fold, 8.8-fold, 9.8-fold, 2.3-fold and 6.3-fold increases were observed for Val, Leu, Ile, Cys and Ser, respectively. Previously, it was found from both experiments and theoretical simulation that the fluorescence behavior of a chromophore attached onto the ionic liquid moiety could be significantly influenced and tuned by the chemical micro-environments created through coupling with different counterions.^19^ Thus, it can be believed that the micro-environment change, as well as the shrinking or swelling of the PIL spheres induced by binding with different amino acids, should be responsible for the observed diverse fluorescence responses of the AIE luminogens integrated in the skeleton of the PIL. Notably, the AIE-based FL channel is more sensitive, due to the intrinsic sensitivity of the fluorescence. The similar structural analytes, for example Val and Leu, which differ only by a single methylene moiety in their backbone chains, could be distinguished by fluorescence. Moreover, isomers Leu and Ile could also be easily discriminated by the FL channel. In our case, to gain richer and more diverse information, the fluorescence enhancements at 515 nm and 554 nm were collected and employed for the discrimination of the amino acids.

**Fig. 5:**
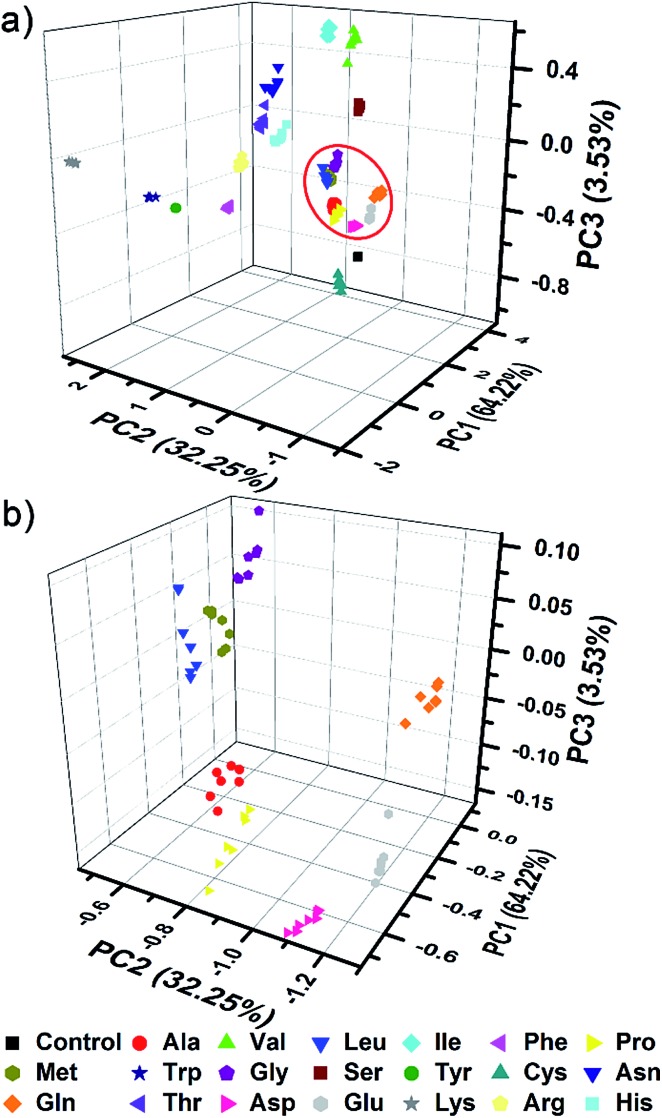
AIE-doped poly(ionic liquid) photonic spheres of the OH^–^ form for the discrimination of 20 natural amino acids at 10 mM. (a) 3D PCA plot; (b) the corresponding magnified plot of the red circle region in (a).

The complex differential information generated from the PhC and FL dual-channels described above was evaluated by using principal component analysis (PCA) for data reduction and classification (3 dimensional information × 21 analytes × 7 trials = a total of 147 samples). As shown in Fig. 5, the resultant 3D PCA score plot intuitively exhibits a clear clustering of all 20 different amino acids, showing the excellent discriminatory power of the single sphere-based AIE-doped photonic sphere. The measured data displayed in a cluster in the PCA plot intuitively reflects the reproducibility or error of the corresponding set of trials. The error can be quantitatively evaluated by the root-mean-square error (RMSE). Additionally, the space distance between the two closely neighboring clusters is indicative of the resolution and this distance can be quantitatively calculated by the Euclidean distance (ED). A bigger distance implies a higher resolution.^1*c*^ Based on linear discriminant analysis (LDA), the leave-one-out validation routine displays 100% accuracy for the classification of all of the 147 samples in our case. The amino acids at lower concentration (1 mM) were also used as targets to test the classification ability of our PIL photonic spheres. Fig. S7† presents the differential responses collected from the PhC and FL channels and Fig. S8† is the obtained PCA plot. Though some clusters in the plot seem a little overlapped due to visual obstruction in the 3D space, the cross validated classification still presents an accuracy of 99%.

### Application in real-world samples and complex mixtures

**Fig. 6:**
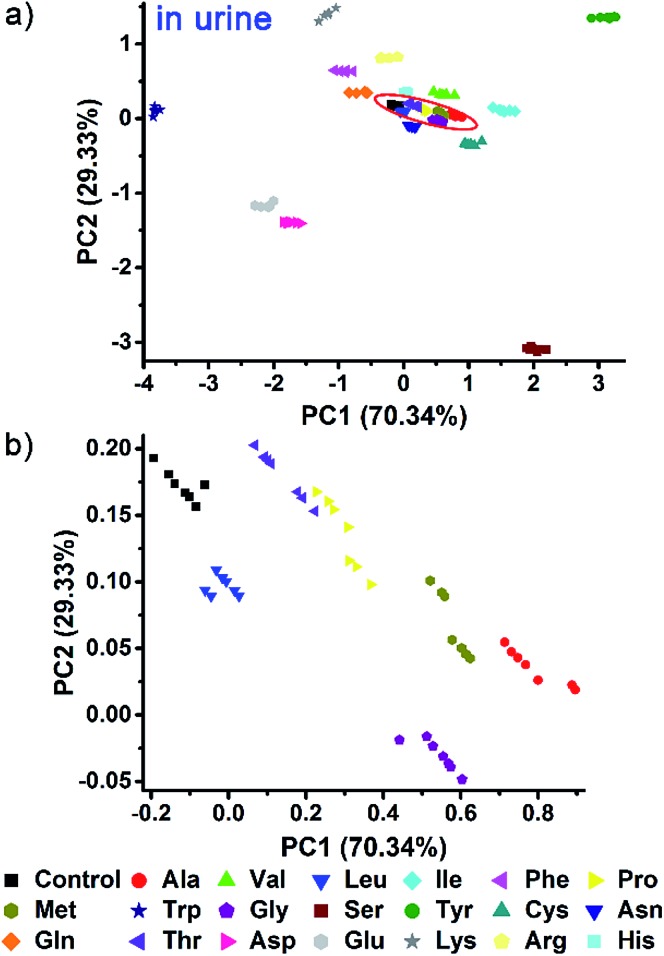
AIE-doped poly(ionic liquid) photonic spheres of the OH^–^ form for the discrimination of 20 natural amino acids at 10 mM in human urine. (a) 2D PCA plot; (b) the corresponding magnified plot of the red circle region in (a).

**Fig. 7:**
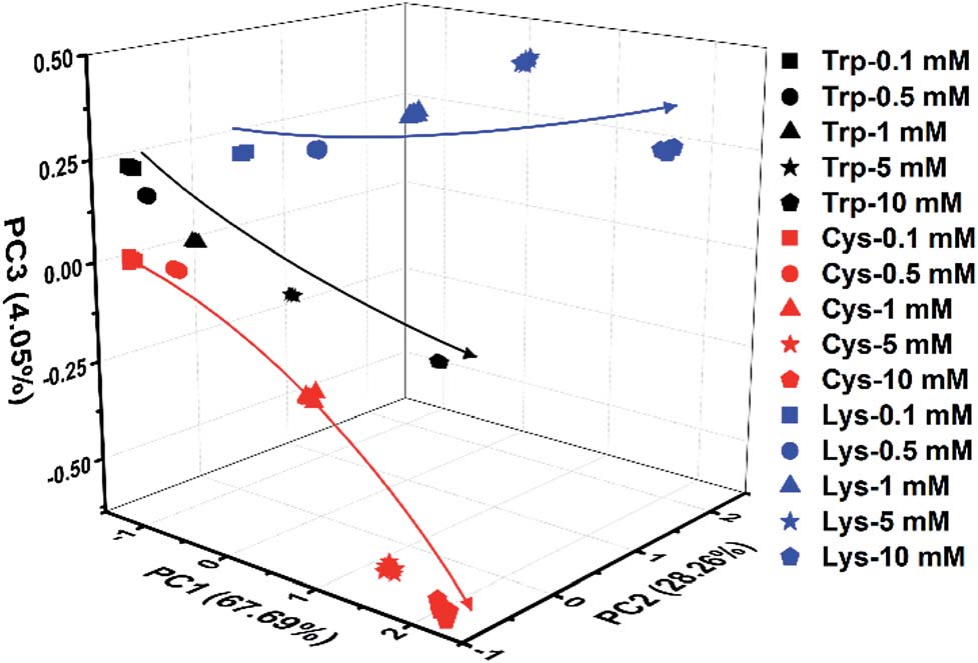
3D PCA plot of the semiquantitative assay of Trp, Cys and Lys at five different concentrations by the AIE-doped PIL photonic spheres of the OH^–^ form.

Indeed, even in more complex physiological environments, our sensing system also exhibits excellent discrimination of the 20 natural amino acids in human urine and 100% correct classification was achieved (Fig. 6). In addition to qualitative identification, a semiquantitative assay is also possible by using our photonic sphere. As shown in Fig. 7, three amino acids, Trp, Cys and Lys, are clearly separated at different gradient concentrations (0.1 mM, 0.5 mM, 1 mM, 5 mM and 10 mM). Each individual series of clusters is distributed along the increasing concentrations. Fig. 7 clearly displays the dependence of the recognition patterns of analytes on their concentrations, indicating that when such a dependence relationship is previously established, the target analytes at different concentrations can be detected and discriminated. In fact, according to the basic principle of the array-based sensing systems, as long as the training data sets are previously established, the sensitizing process is still precise when the concentration of a sample is unknown.^1*c*^

**Fig. 8:**
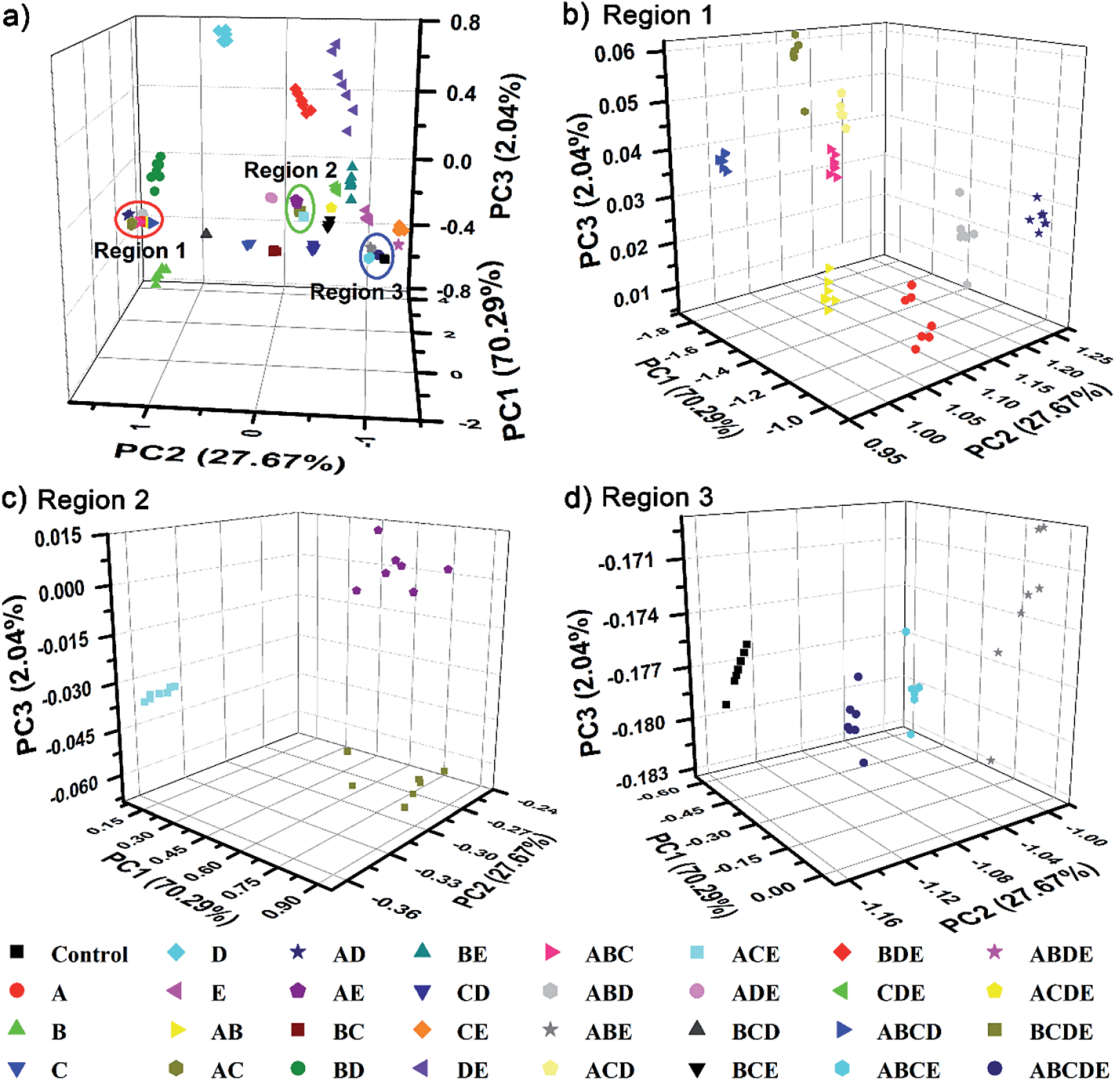
AIE-doped poly(ionic liquid) photonic spheres of the OH^–^ form for the discrimination of 1 control analyte, 5 unitary analytes and 26 mixture analytes at 10 mM for all of the amino acids. (A–E) represent Lys, Cys, Trp, Ile and Glu, respectively. (a) 3D PCA plot; (b) corresponding magnified plot of the red circle region 1 in (a); (c) corresponding magnified plot of the green circle region 2 in (a) and (d) corresponding magnified plot of the blue circle region 3 in (a).

To further demonstrate the discrimination power, as well as the practical value of our single sphere-based PIL sphere, a series of mixtures of amino acids as target analytes was investigated. Five typical amino acids, Lys, Cys, Trp, Ile and Glu, were selected and named as A, B, C, D and E, respectively. A total of all 26 possible equimolar mixtures of A, B, C, D and E were prepared and explored, including 10 binary systems (AB, AC, AD, AE, BC, BD, BE, CD, CE and DE), 10 ternary systems (ABC, ABD, ABE, ACD, ACE, ADE, BCD, BCE, BDE and CDE), 5 quaternary systems (ABCD, ABCE, ABDE, ACDE and BCDE) and 1 quinary system (ABCDE). Surprisingly, Fig. S9† displays quite rich PhC and FL signal changes of the PIL spheres when they were incubated in mixtures of amino acids. Based on the differential information collected from a single PIL sphere, the well-separated clusters in the 3D PCA plot (Fig. 8) clearly confirm the successful classification of the used multi-analyte systems (5 unitary analytes, 26 mixture analytes and 1 control analyte) with 100% correct identification. Additionally, it is well-known that differential sensing is capable of predicting unknown samples when they have previously been used as training data sets.^1*c*^ In this work, 15 blind samples from the 32 training analytes in Fig. 8 were also correctly identified (Table S1†). This preliminary result indicates that, as long as the training sets are appropriately established, it is possible for our sensing system to predict the components or ratios of different compounds in mixtures.

The identification of natural amino acids is biologically important but intrinsically challenging, especially when using a simple and feasible method to achieve this tough goal. In the past few decades, although the detection of individual or several specific amino acids has made great advancements,^20,21^ to the best of our knowledge, there are only three reports from Severin,^22*a*^ Lin^22*b*^ and the Song group^22*c*^ describing the detection of all 20 amino acids. In their studies, cross-reactive arrays with about ten sensing elements are generally required and at the same time the performed detections are usually accompanied with a complicated operation procedure and low throughput. In our case, however, a single sphere is enough for implementing the same sensing purpose, even for broader mixture systems which have never been reported, and the experimental procedures used are greatly simplified. It should be noted that the sensor arrays reported so far can only be applied to identify mixtures with large differences in their components.^23^ Any change of the target mixture, such as slightly adjusting the relative proportions of the components or adding new components, may cause trouble for the discrimination. None of the reported arrays demonstrated the sensing of all 26 possible equimolar mixtures of five components, as in our case.

### Customizable arrays for target-oriented discrimination

**Fig. 9:**
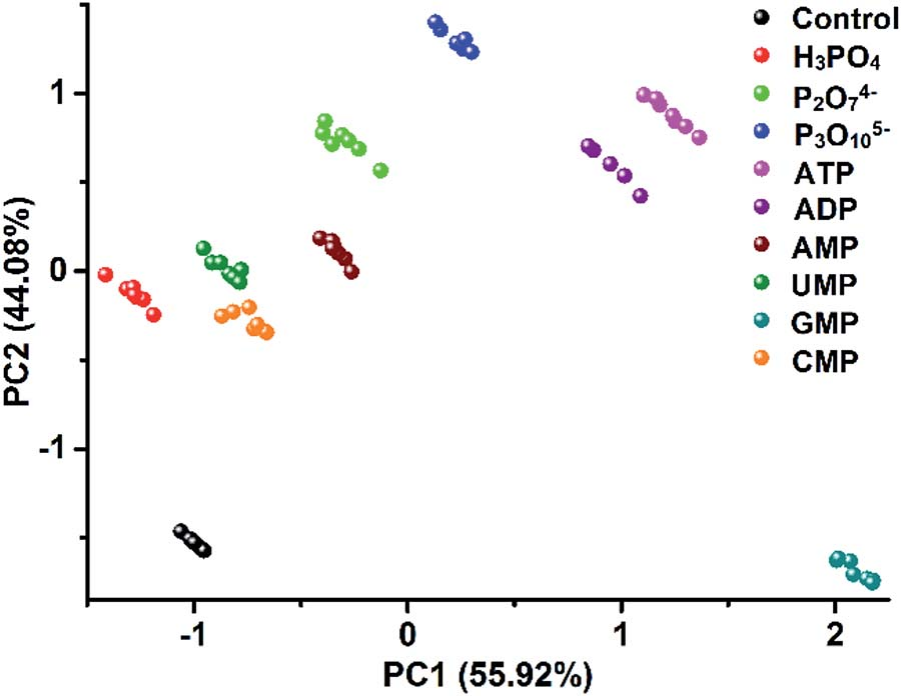
2D PCA plot of the AIE-doped PIL photonic spheres (OH^–^ form) for the discrimination of nine phosphate derivatives at 10 μM.

The unique multiple intermolecular interaction feature enables ILs to strongly interact with different classes of compounds and thus to have strong solvation power. Owing to this beneficial property, ILs have already been demonstrated to be used as a universal solvent for different classes of materials,^10^ even for tough celluloses.^24^ In our work, due to this universal structural feature, we also found that the created PIL spheres could actually be used as a multi-channel sensing platform to effectively detect a broader class of analytes, not only for amino acids but also for other classes of multi-analytes. As a demonstration, the discrimination of nine biologically important phosphate derivatives (Fig. S10†) was chosen as another challenge system for our study, including phosphoric acids bearing different nucleobases (AMP, UMP, GMP and CMP) and different numbers of phosphate groups (AMP, ADP and ATP), as well as inorganic phosphates (phosphate, pyrophosphate and triphosphate). Fig. S11† shows the differential information collected from the dual optical signal channels of the PIL spheres as mentioned above. In Fig. 9, all of the nine phosphate derivatives are well clustered into nine different groups and a jackknifed matrix with cross-validation reveals a discrimination accuracy of 100% using this “lab-on-a-sphere” paradigm. Compared to the reported arrays for phosphate derivatives, our single sphere-based array shows prominent advantages.^25,26^

**Fig. 10:**
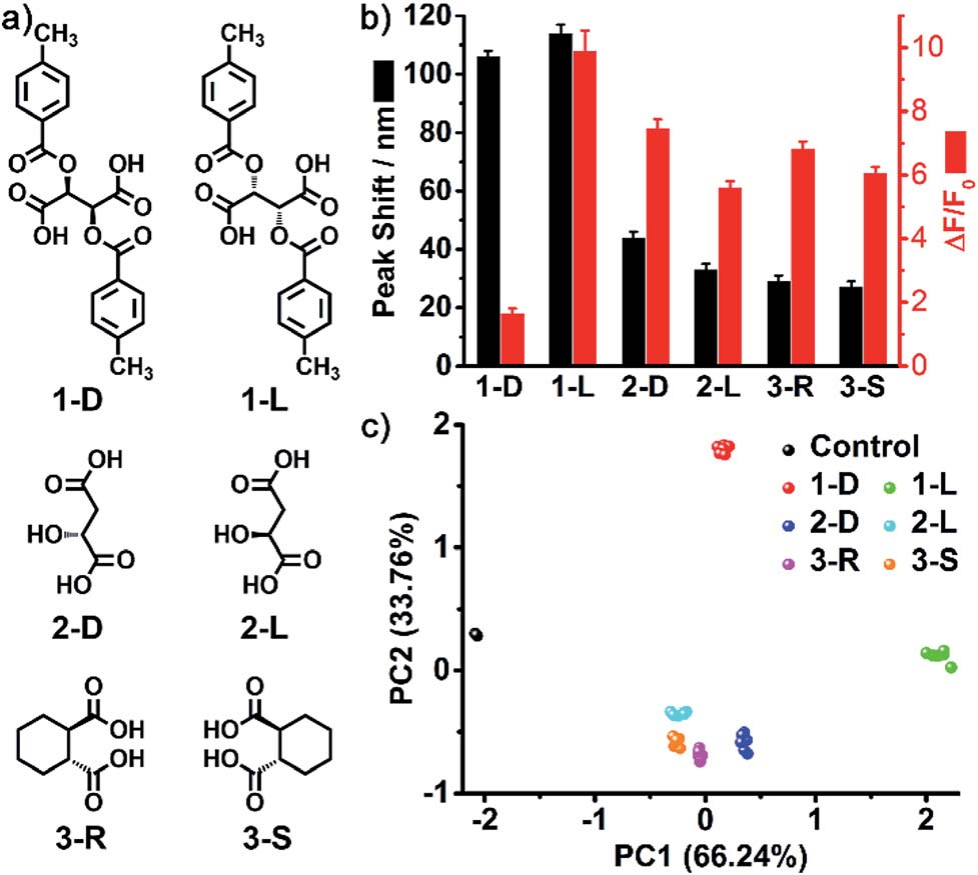
(a) Chemical structures of the three pairs of dicarboxylic acid enantiomers. (b) Responses of the AIE-doped poly(ionic liquid) photonic spheres (chiral spiral borate form) to the three pairs of dicarboxylic acid enantiomers at 10 mM. (c) 2D PCA plot of the AIE-doped poly(ionic liquid) photonic spheres (chiral spiral borate form) for the discrimination of the six chiral vicinal dicarboxylic acids at 10 mM. Here 1d/l, 2d/l and 3*R*/*S* represent di-*p*-toluoyl-d/l-tartaric acid, d/l-malic acid and (1*R*,2*R*)/(1*S*,2*S*)-cyclohexane-1,2-dicarboxylic acid, respectively.

**Fig. 11:**
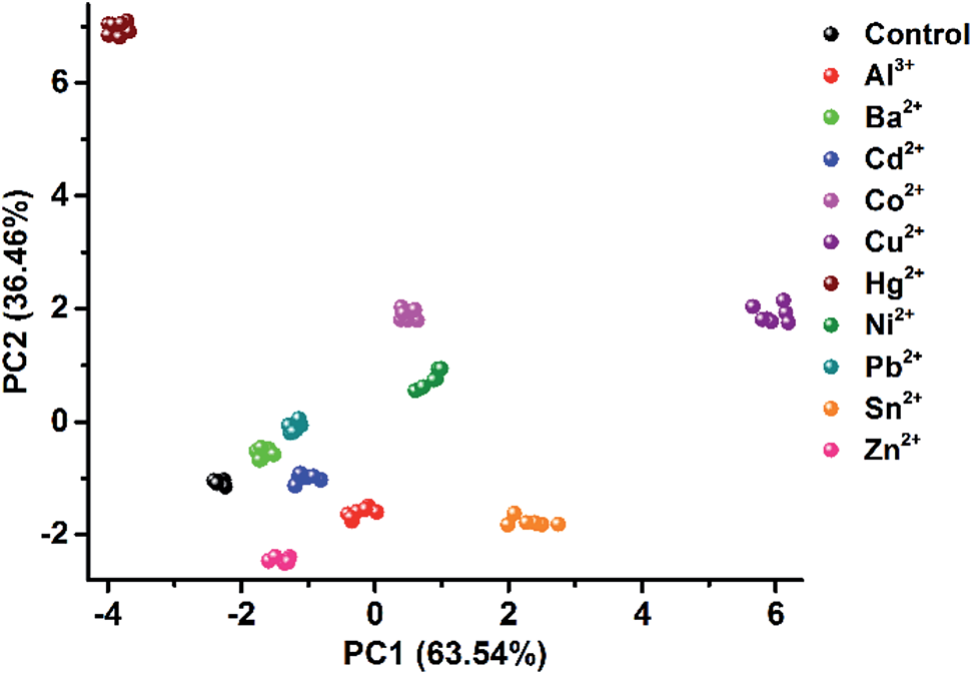
2D PCA plot of the AIE-doped PIL photonic spheres (citrate form) for the discrimination of ten metal ions at 100 μM.

As one of the most distinct features of ILs, counterion exchange provides a very efficient and convenient way to access various ILs with tunable physicochemical properties. This beneficial feature allows for the rapid establishment of a library of sensing elements as well as for the facile modulation and high-throughput screening of the required sensing performance. More attractively, when functional counterions are employed, the ILs could be designed or customized for a specific task. Based on this “task-specific” concept,^15^ we also found that the prepared photonic spheres could be biased towards a particular class of analytes by using selective receptors as counteranions. As an exemplification, in this work, the AIE-doped PIL photonic spheres with chiral spiral borate as the counteranion were firstly constructed through an anion exchange of the prepared parent PIL spheres (with Br^–^ as the anion) with a spiral borate salt (Fig. S12†) and used for the identification of three pairs of dicarboxylic acid enantiomers (Fig. 10a). Based on the differential response information obtained from the PhC and FL channels (Fig. 10b), the PCA results are presented in Fig. 10c. As expected, the three classes of chiral dicarboxylic acids could be excellently detected and discriminated. Importantly, besides the chemical selectivity, the enantioselective discrimination between a pair of enantiomers was also well achieved.^27^ As a control experiment, the parent PIL spheres (with Br^–^ as the anion) were used for identifying the dicarboxylic acids mentioned above. The obtained results reveal that the incorporated chiral counteranion is important for the observed enantiomeric recognition ability of the PIL spheres. In fact, a similar observation was also found for the detection of multi-metal ions.^28^ When citrate, which is a very good chelating agent for metal ions, was used as the counteranion (Fig. S13†), the resultant PIL photonic sphere became an excellent sensing platform for metal ions, as shown in Fig. 11.

**Chart 1:**
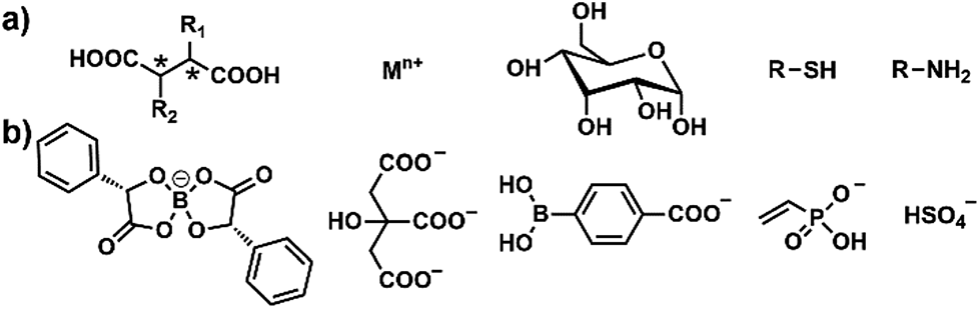
(a) Five representative classes of targeted multi-analytes including chiral dicarboxylic acids, metal ions, sugars, biothiols and bioamines. (b) Corresponding customizable counteranions for target-oriented sensing, including the selective recognition of chiral dicarboxylic acids by a chiral spiral borate anion, of metal ions by citrate, of sugars by phenyl boronic acid, of biothiols by a vinyl anion, and of bioamines by a Lewis acid.

In our recent work, several other functional counterions (Chart 1) were successfully introduced into our PIL spheres by simple anion exchange reactions. The preliminary sensing results are very promising. All of these PIL spheres could be facilely biased for implementing a specific task on demand, for example for the identification of sugars, biothiols or bioamines. All of the results indicate that the described PIL spheres are highly designable, customizable and exhibit extraordinary extendibility. Notably, the unique multiple intermolecular interaction feature of the IL units imparts our PIL spheres with high cross-reactivity upon interaction with different analytes, either of the same class or not, while functional counteranions with selective recognition capability make the PIL spheres customizable for different classes of analytes, thus offering our spheres the concept of “selective array-based sensing”.^29^

## Conclusions

The tendency of sensing device miniaturization necessitates the creation of an efficient array system with a minimum number of sensing elements. Multidimensional sensing devices based on a molecule or a particle represent a very promising concept with numerous advantages for multi-analyte identification, such as miniaturizing the sensing device, simplifying its implementation, enhancing its differential efficiency and in particular promoting its development in high-throughput screening.^2,30^ Although great progress has been made over the past few years, the facile construction of such a sensing system from a single material is still a big challenge, in particular for developing one system with a customizable feature for the target-oriented discrimination of a broad spectrum of multi-analytes. In this work, based on an AIE-doped poly(ionic liquid) photonic sphere, a novel single sphere-based multi-channel sensing system with a series of advantages was developed. By simultaneously exploiting the unique properties of ionic liquids, AIE luminogens and photonic structures, such a single-sphere system can discriminate broader classes of multi-analytes on-demand and exhibit excellent discriminatory power, enhanced selectivity, high flexibility and great extendibility. The chosen model recognition systems above clearly highlight the significant value of our customizable AIE-doped photonic sphere as a target-oriented sensing platform for multi-analyte detection. Furthermore, the successful differential sensing of all 20 amino acids in human urine and their 26 unprecedented complex mixtures demonstrates the tremendous potential of our sphere system for practical application. More importantly, by doping differently colored AIE luminogens the single-sphere strategy can be facilely extended to produce higher-level multi-channel signals. On the other hand, by co-polymerizing multiple functional ionic liquids with different counteranions, the obtained multi-component sphere can acquire more remarkable diversity and sensitivity upon interacting with analytes. These two proposed strategies will be very useful in the future for precisely identifying more complicated multi-analyte systems, especially in real-life samples.

## Live subject statement

The human urine related tests in this work were performed in compliance with the relevant laws and institutional guidelines for the Academic Norms System established by the Academic Committee of Tsinghua University. This committee have approved the experiments and informed consent from the volunteer who provided the urine samples was obtained for experimentation with human subjects.
