# Supplementary material for: AIE-doped poly(ionic liquid) photonic spheres: a single sphere-based customizable sensing platform for the discrimination of multi-analytes
Source: Chem Sci. 2017 Jun 30;8(9):6281–9. doi: 10.1039/c7sc02409f (PMC5628402; doi:10.1039/c7sc02409f)
Supplement: Supplementary file 1 [file SC-008-C7SC02409F-s001.pdf]

Electronic Supplementary Material (ESI) for Chemical Science.
This journal is © The Royal Society of Chemistry 2017

Electronic Supplementary Information

AIE-Doped Poly(ionic liquid) Photonic Sphere: A Single-Sphere Based Customizable Sensing Platform for Discrimination of Multianalytes

Wanlin Zhang,[a]‡ Ning Gao,[a]‡ Jiecheng Cui,[a] Chen Wang,[a] Shiqiang Wang,[a] Guanxin Zhang,[b] Xiaobiao Dong,[b] Deqing Zhang,[b]* and Guangtao Li[a]*

[a]Department of Chemistry, Key Lab of Organic Optoelectronics and Molecular Engineering, Tsinghua University, Beijing 100084, P. R. China

[b]Institute of Chemistry, Chinese Academy of Sciences, Beijing 100190, P. R. China

‡Co-first authors: Wanlin Zhang; Ning Gao

*Corresponding authors:
Guangtao Li (lgt@mail.tsinghua.edu.cn); Deqing Zhang (dqzhang@iccas.ac.cn)

Contents

General...S2
Part 1 preparation of parent AIE-doped poly(ionic liquid) photonic spheres...............................S3
Synthesis of ionic liquid monomer, crosslinker and AIE luminogen...S3
Preparation of parent spheres...S5
Part 2 spheres of OH^- form for the discrimination of amino acids and their mixtures....................S9
Preparation of spheres of OH^- form...S9
Spheres of OH^- form for the discrimination of 20 natural amino acids...S9
Spheres of OH^- form for the discrimination of mixtures of amino acids...S13
Part 3 spheres of OH^- form for the discrimination of phosphate derivatives.................................S15
Part 4 customizable spheres of CSBA form for the discrimination of chiral dicarboxylic acids..S16
Part 5 customizable spheres of citrate form for the discrimination of metal ions.........................S17
References...S17

General

Commercially available reagents and solvents were used as purchased from the chemical suppliers. Deionized water, 2-[4-(2-hydroxyethyl)-1-piperazinyl]ethanesulfonic acid buffer (HEPES, pH = 7.2) and human urine from volunteers were used for the preparation of target analyte solutions. ^{1}H and ^{13}C NMR spectra were recorded on a 400 MHz NMR spectrometer (JEOL, ECS-400). Electrospray ionization mass spectrometry (ESIMS) was measured on a mass spectrometer (Bruker, Esquire-LC). The fluorescence measurements of AIE activity were carried out using a fluorescence spectrometer (Perkin-Elmer, LS55). The optical images of spheres were recorded by an optical microscope (OLYMPUS, 51M) equipped with a CCD camera (OLYMPUS, UTV0.5XC-3). The fluorescence images of spheres were taken using an inverted fluorescence microscope (OLYMPUS, IX71; excitation filter 330-385 nm; long-pass emission > 420 nm) equipped with a CCD camera (OLYMPUS, DP73). The reflection spectra and emission spectra of spheres were measured by a microscope equipped with a fiber optic spectrometer (Ocean Optics, USB2000+). The size and structure of nanoparticles and spheres were characterized using scanning electron microscope (SEM) (Hitachi, SU8010). Energy-dispersive X-ray spectroscopy (EDX) measurements were performed with the spectrometer attached on SEM system. The FTIR spectra of spheres were obtained with an attenuated total reflection IR spectroscopy (Bruker, VERTEX70). The PCA and cross-validated LDA (leave-one-out) were performed using Matlab software.

Part 1 preparation of parent AIE-doped poly(ionic liquid) photonic spheres

Synthesis of ionic liquid monomer, crosslinker and AIE luminogen

Imidazolium-based ionic liquid monomer (IL monomer, i.e., 1-propyl-3-vinylimidazolium bromide), crosslinker (IL crosslinker, i.e., 1,6-di(3-vinylimidazolium) hexane bromide) and AIE luminogen were synthesized.

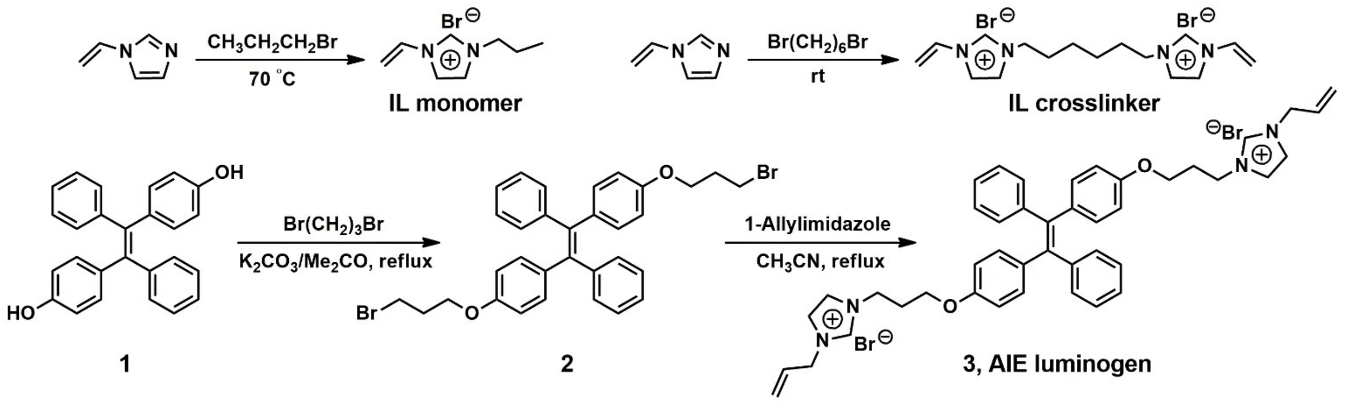

Fig. S1 Synthetic routes to imidazolium-based ionic liquid monomer, crosslinker and AIE luminogen.

Synthesis of IL monomer. IL monomer was prepared according to reported procedures in literature.[1] ^{1}H NMR (400 MHz, DMSO): *δ* 9.52 (1H, s), 8.21 (1H, s), 7.94 (1H, s), 7.30 (1H, dd, *J* = 8.8 Hz, *J* = 15.6 Hz), 5.96 (1H, dd, *J* = 2.3 Hz, *J* = 15.7 Hz), 5.43 (1H, dd, *J* = 2.3 Hz, *J* = 8.7 Hz), 4.17 (2H, t, *J* = 7.1 Hz), 1.91-1.79 (2H, m), 0.88 (3H, t, *J* = 7.4 Hz).

Synthesis of IL crosslinker. IL crosslinker was prepared according to reported procedures in literature.[2] ^{1}H NMR (400 MHz, DMSO): *δ* 9.72 (2H, s), 8.26 (2H, s), 8.01 (2H, s), 7.34 (2H, dd, *J* = 8.8 Hz, *J* = 15.7 Hz), 6.00 (2H, dd, *J* = 2.3 Hz, *J* = 15.6 Hz), 5.42 (2H, dd, *J* = 2.3 Hz, *J* = 8.8 Hz), 4.23 (4H, t, *J* = 7.2 Hz), 1.82-1.87 (4H, m), 1.31 (4H, t, *J* = 6.8 Hz).

Synthesis of compound 2. Compound **1** was prepared according to reported procedures in literature.[3] A mixture of compound **3** (364.1 mg, 1 mmol), 1,3-dibromopropane (807.5 mg, 4 mmol) and potassium carbonate (1.105 g, 8 mmol) in acetone (20 mL) was refluxed for 12 hours under nitrogen. After cooling to room temperature, the solution was filtered, and the filtrate was evaporated under reduced pressure. The residue was dissolved in CH_2Cl_2 and subjected to column chromatography with EA (ethyl acetate)/PE (petroleum ether, 60-90 ^oC) (v/v, 1/50) as eluent. Compound **2** was obtained as green oil (444.6 mg, 0.73 mmol) in 73.3% yield. ^{1}H NMR (400 MHz, $CDCl_3$): *δ* 7.83 (4H, d, *J* = 8.8 Hz), 7.76 (4H, d, *J* = 7.2 Hz), 7.59-7.55 (2H, m), 7.49-7.46 (4H, m), 6.97 (4H, d, *J* = 8.8 Hz), 4.20 (4H, t, *J* = 5.8 Hz), 3.64-3.61 (4H, t, *J* = 5.8 Hz), 2.39-2.33 (4H, m). ^{13}C NMR (100 MHz, $CDCl_3$): *δ* 156.1, 143.2, 143.1, 138.7, 135.7, 135.6, 131.5, 131.4, 130.4, 126.7, 126.5, 125.2, 112.7, 112.6, 64.2, 31.5, 29.0. MS: calcd. for $C_{32}H_{30}Br_2O_2$: 606.5; found: 606.3.

Synthesis of compound 3. A mixture of compound **2** (303.0 mg, 0.5 mmol) and 1-allylimidazole (216.3 mg, 2 mmol) in CH_3CN (20 mL) was refluxed for 24 hours. After cooling to room temperature, the solvent was evaporated under reduced pressure. The residue was dissolved in CH_2Cl_2 and subjected to column chromatography with CH_2Cl_2/CH_3OH (v/v, 5/1) as eluent. Compound **3** was obtained as green oil (222.0 mg, 0.27 mmol) in 54.1% yield. ^{1}H NMR (400 MHz, CD_3OD): *δ* 9.03 (2H, s), 7.69 (2H, s), 7.60 (2H, s), 7.09 (6H, s), 6.99 (4H, d, *J* = 7.5 Hz), 6.88 (4H, d, *J* = 8.7 Hz), 6.60 (4H, d, *J* = 8.4 Hz), 6.09-5.95 (2H, m), 5.42-5.36 (4H, m), 4.81 (4H, d, *J* = 6.2 Hz), 4.43 (4H, t, *J* = 6.6 Hz), 3.98 (4H, t, *J* =

5.4 Hz), 2.35-2.29 (4H, m). ^{13}C NMR (100 MHz, CD_3OD): δ 156.9, 144.1, 139.9, 136.6, 136.1, 132.2, 131.0, 130.5, 129.3, 128.2, 127.7, 127.4, 126.0, 125.3, 122.8, 122.4, 120.8, 113.4, 64.4, 51.3, 29.2. HR-ESI: calcd. for $C_{44}H_{46}N_4O_2^{2+}$ $(m/z)^+$: 331.18054; found: 331.18049.

AIE activity of compound 3

The synthesized compound **3** here, an imidazolium-bearing tetraphenylethylene derivative, shows typical AIE activity in Figure S2 as reported for other tetraphenylethylene molecules. The solutions of compound **3** in good solvents such as CH_2Cl_2 are almost non-emissive. The fluorescence of compound **5** can be turned on after aggregation induced by addition of poor solvents such as hexane into CH_2Cl_2. At 90 vol% hexane content, the fluorescence intensity is 53-fold higher than that of pure CH_2Cl_2 solution, while the emission maximum is still located at 475 nm. Therefore, the synthesized compound **3** can be used as AIE luminogen.

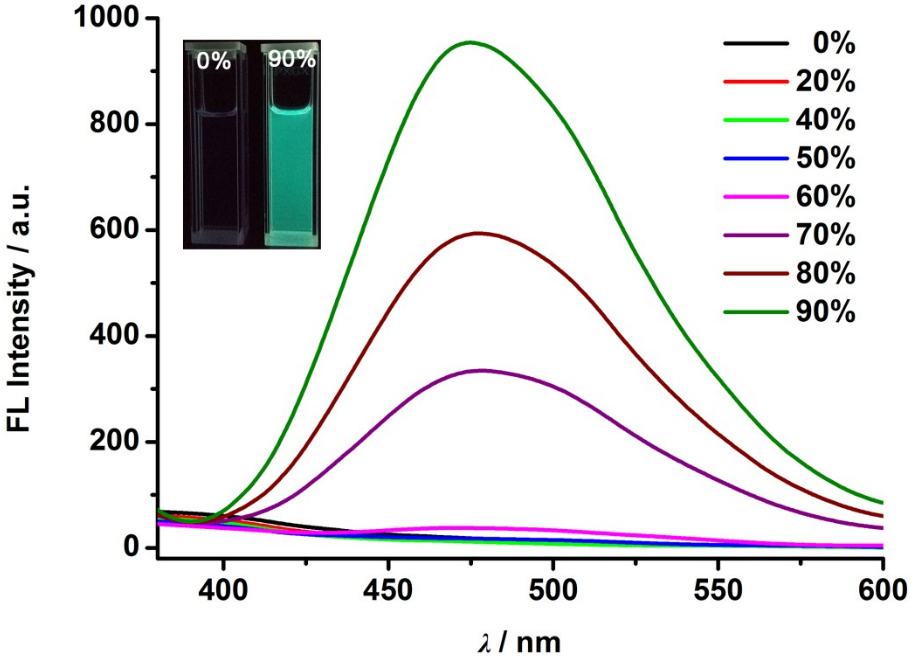

Fig. S2 Fluorescence spectra of compound **3** (10 μM) in the hexane/CH_2Cl_2 mixtures with different volume fractions of hexane, λ_{ex} = 335 nm. Insets are the fluorescence photographs of solution of compound **3** in CH_2Cl_2 (left) and in 90% hexane/CH_2Cl_2 mixture (right) under illumination of a handheld UV (365 nm) lamp.

Preparation of parent spheres

Parent AIE-doped PIL photonic spheres were prepared using an analogous method previously reported by our group.[4] These spheres were also fabricated by using a two-step method. First, monodisperse silica nanoparticles with a diameter of 170 nm self-assembled into ordered lattices by droplet-based microfluidics. Monodisperse silica particles with a diameter of ca. 170 nm were synthesized by the modified Stöber method.[5] Second, the solution containing 0.60 g ionic liquid monomer, 0.036 g crosslinker, 200 μL AIE luminogen stock methanol solution (3 mM) and 10 μL photoinitiator was infiltrated into the void spaces of the photonic spheres by capillary force. After polymerization under UV radiation followed by removal of silica template, the resultant parent spheres with highly ordered 3D inverse opal structure and doped AIE luminogen were fabricated.

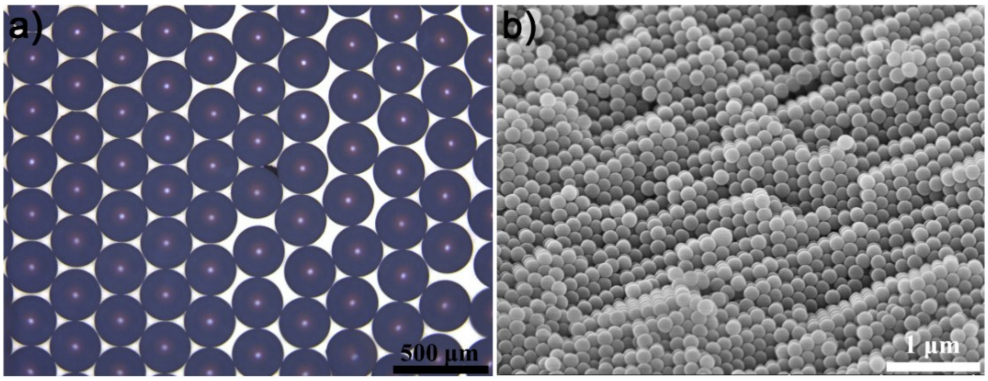

Fig. S3 a) Optical image of silica colloidal crystal spheres; b) SEM image of silica colloidal crystal spheres inside.

¹H NMR spectra of IL monomer and crosslinker

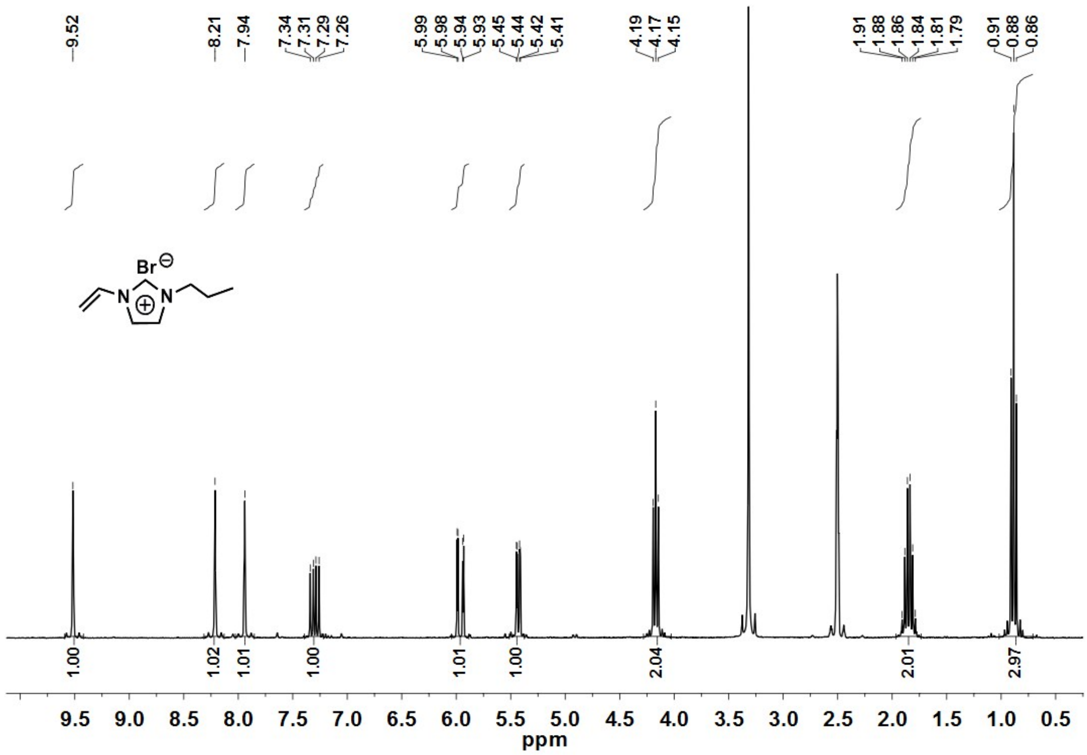

¹H NMR spectrum of IL monomer in DMSO.

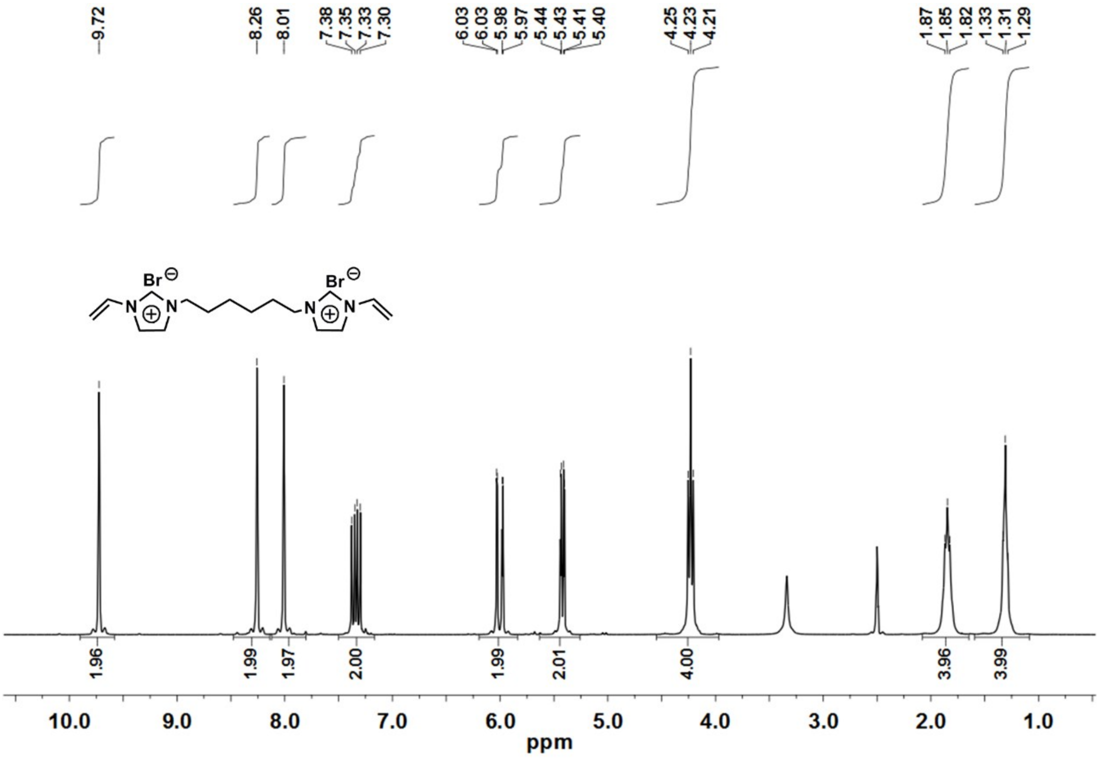

¹H NMR spectrum of IL crosslinker in DMSO.

^{1}H NMR and ^{13}C NMR spectra of compound 2

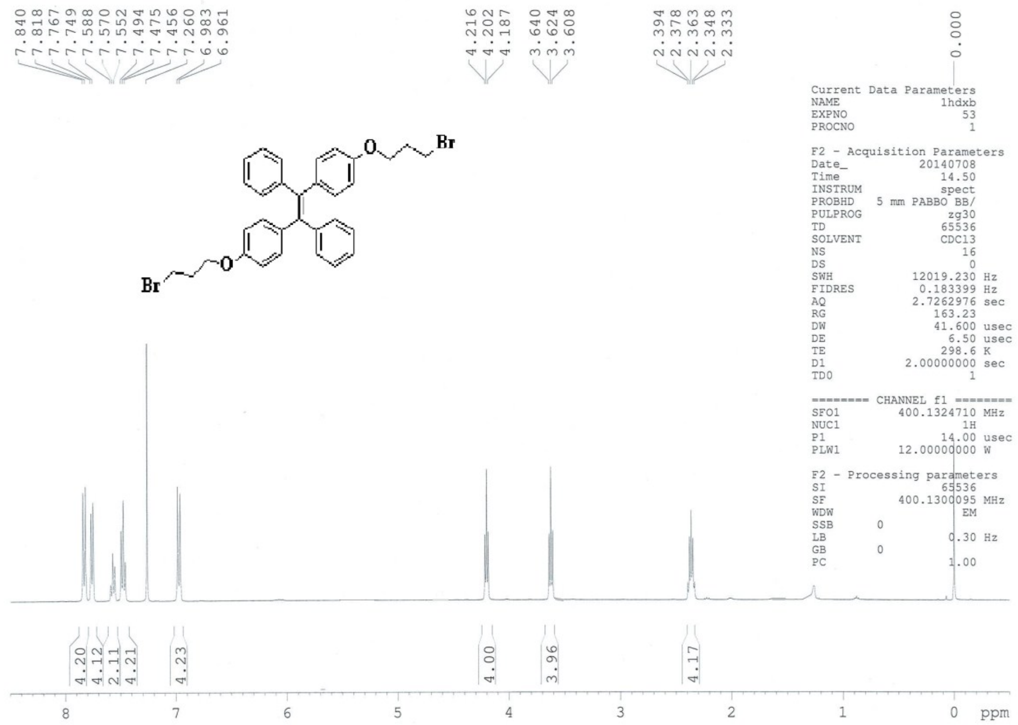

^{1}H NMR spectrum of compound **2** in $CDCl_3$.

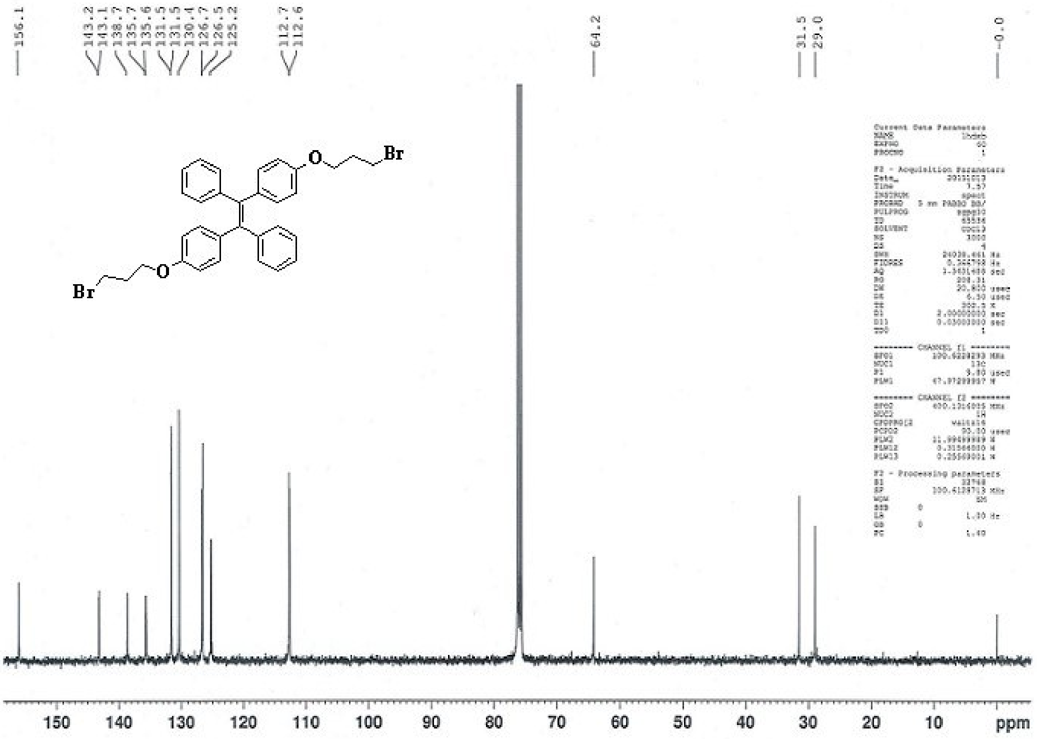

^{13}C NMR spectrum of compound **2** in $CDCl_3$.

^{1}H NMR and ^{13}C NMR spectra of compound 3

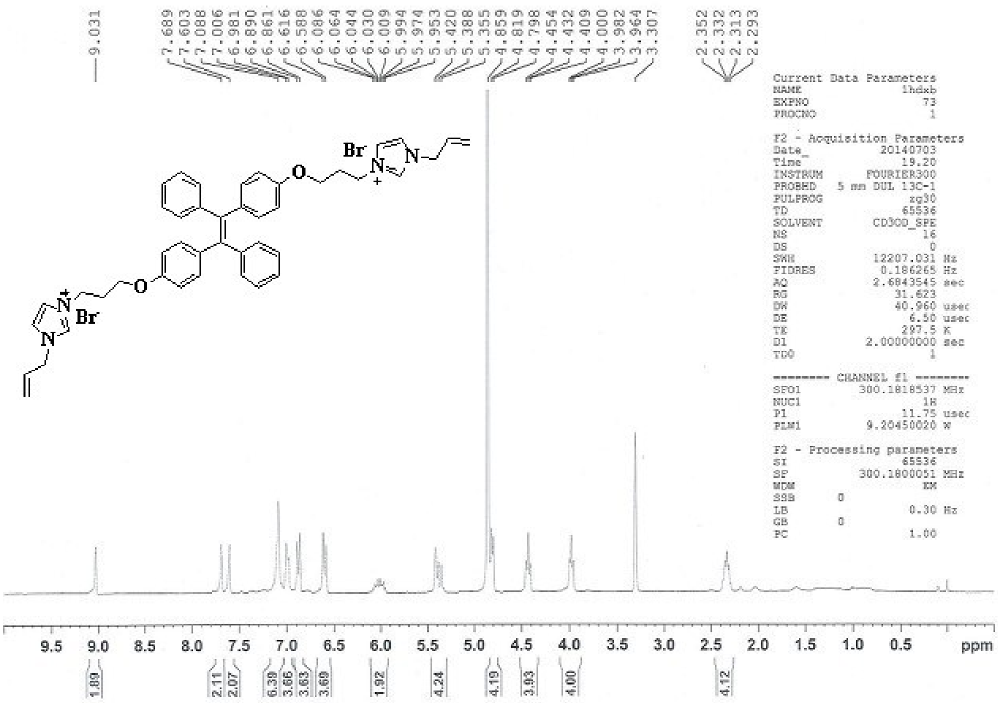

^{1}H NMR spectrum of compound **3** in CD_3OD.

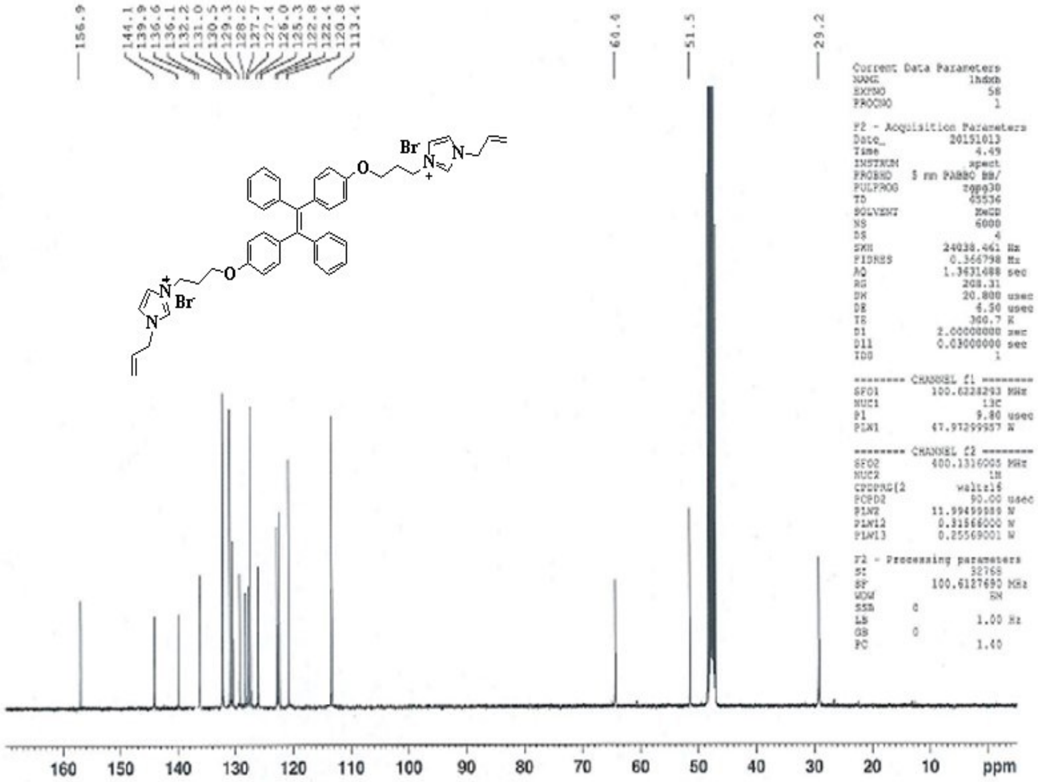

^{13}C NMR spectrum of compound **3** in CD_3OD.

Part 3 Spheres of OH^- form for the discrimination of amino acids and their mixtures

Preparation of spheres of OH^- form

Counteranions of the parent spheres were completely converted from Br^- to OH^- by virtue of facile anion exchange according to a reported method in literature.[6] The above parent AIE-doped PIL photonic spheres were soaked in N_2-saturated 1 M KOH aqueous solution for 24 h to convert the counteranions from the form of Br^- to OH^-. This procedure was repeated three times to ensure thorough conversion of counteranions. Afterwards, the converted spheres were washed by N_2-saturated deionized water until the pH of residual water was neutral. Thus, spheres of OH^- form for target multianalytes (amino acids and phosphate derivatives) were prepared. Remarkably, a very significant Bragg diffraction wavelength red-shift up to 157 nm was observed after the anion replacement, accompanying with a distinct color change of spheres from cyan to red by naked eye (Figure S5a). Energy-dispersive X-ray spectra further confirmed the complete conversion of Br^- to OH^- since no Br could be detected after the anion conversion (Figure S5b).

Spheres of OH^- form for the discrimination of 20 natural amino acids

AIE-doped PIL photonic spheres of OH^- form were incubated in 1 mL amino acid solutions for three days at room temperature under continuous stirring on an orbital shaker to achieve reaction equilibrium. Each sensing response to an analyte was measured with seven individual spheres to test reproducibility. Then the optical images, fluorescence images, reflection spectra and fluorescence spectra of spheres after binding with analytes were recorded. It should be noted that reflection spectra and emission spectra could be easily achieved on a microscope equipped with a fiber optic spectrometer in different detection modes. Bragg diffraction peak shifts ($\Delta\lambda$) were obtained by peak position before binding (λ_0) subtracting peak position after binding (λ_1): $\Delta\lambda = \lambda_0 - \lambda_1$. The folds of fluorescence enhancement $\Delta F/F_0$ at 515 nm (or at 554 nm) were calculated by attaining fluorescence intensity before (F_0) and after (F_1) binding with amino acids at 515 nm (or at 554 nm): $\Delta F/F_0 = F_1/F_0 - 1$. Then, the data was processed using PCA and LDA by Matlab.

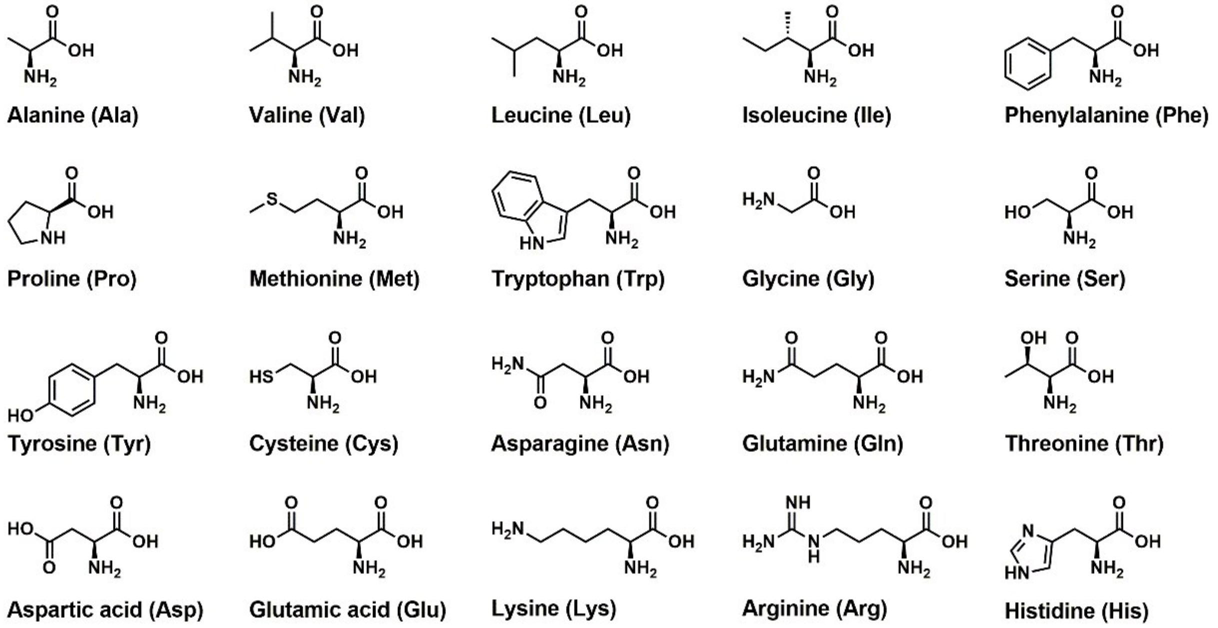

Fig. S4 Chemical structures and name abbreviations of 20 amino acids.

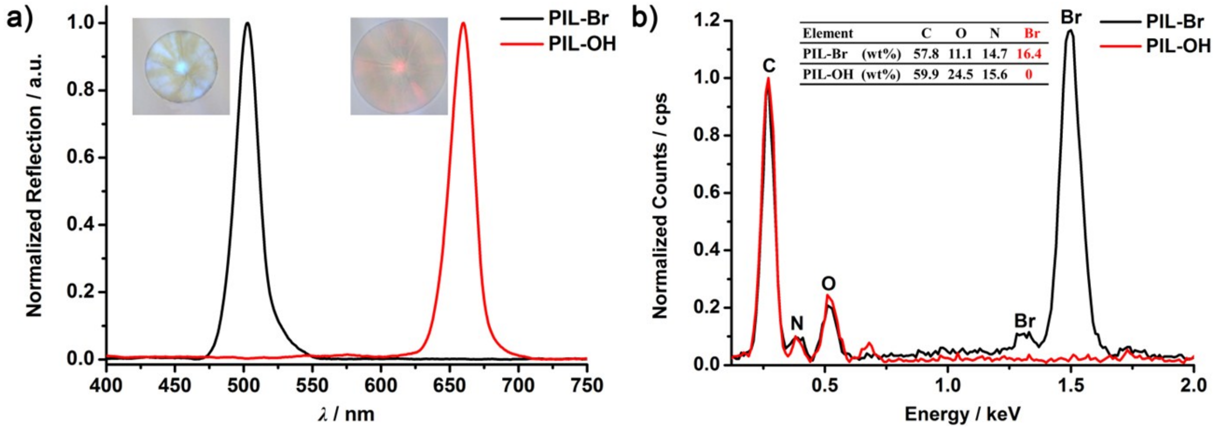

Fig. S5 a) Reflection spectra and corresponding inserted optical images of PIL-Br and PIL-OH; b) EDX spectra of PIL-Br and PIL-OH. PIL-Br and PIL-OH represent PIL photonic spheres of Br^- and OH^- form.

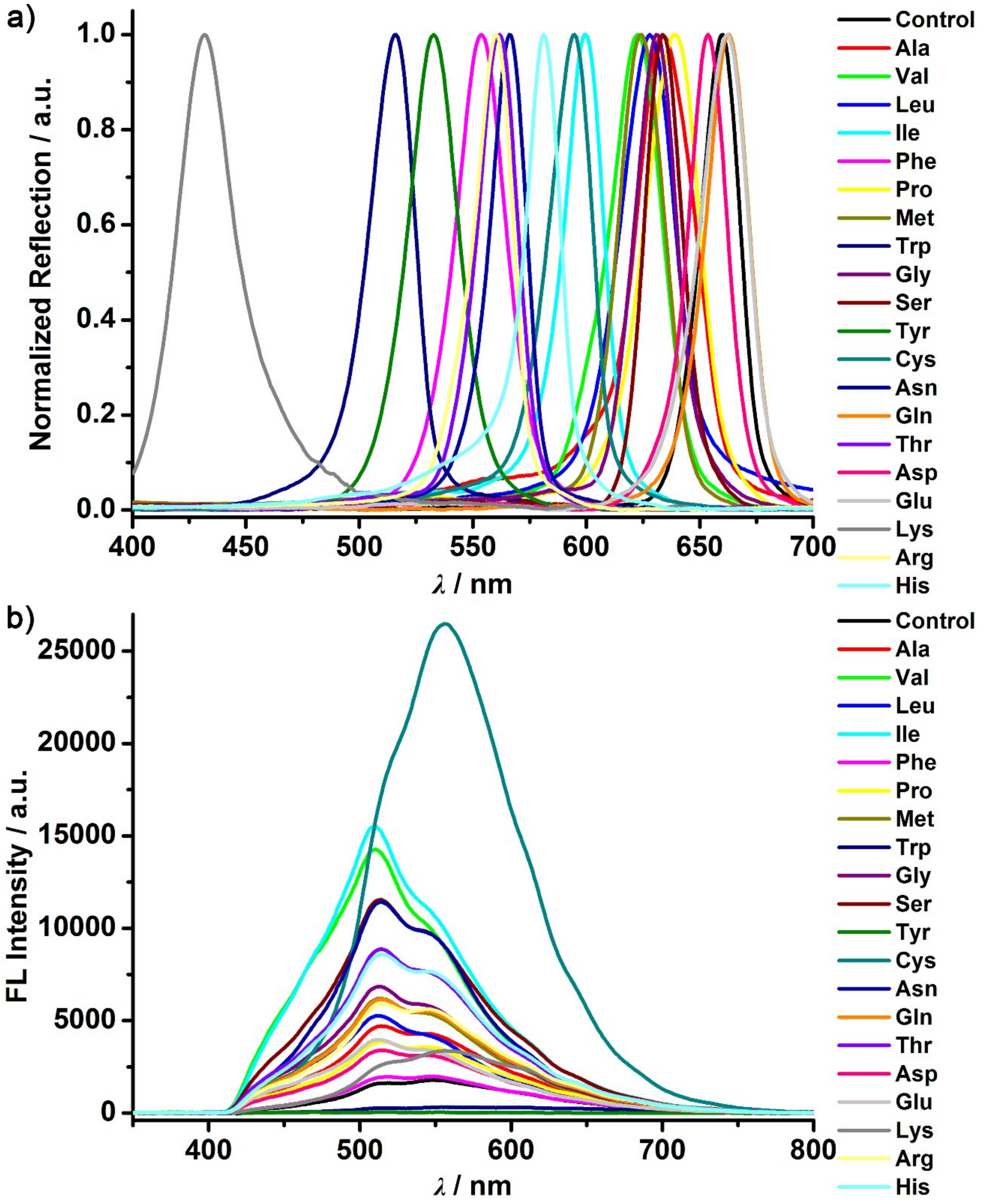

Fig. S6 Responses of AIE-doped PIL photonic spheres (OH^- form) to 20 natural amino acids at 10 mM: a) reflection spectra; b) emission spectra. The integration time for recording FL spectra is 200 ms.

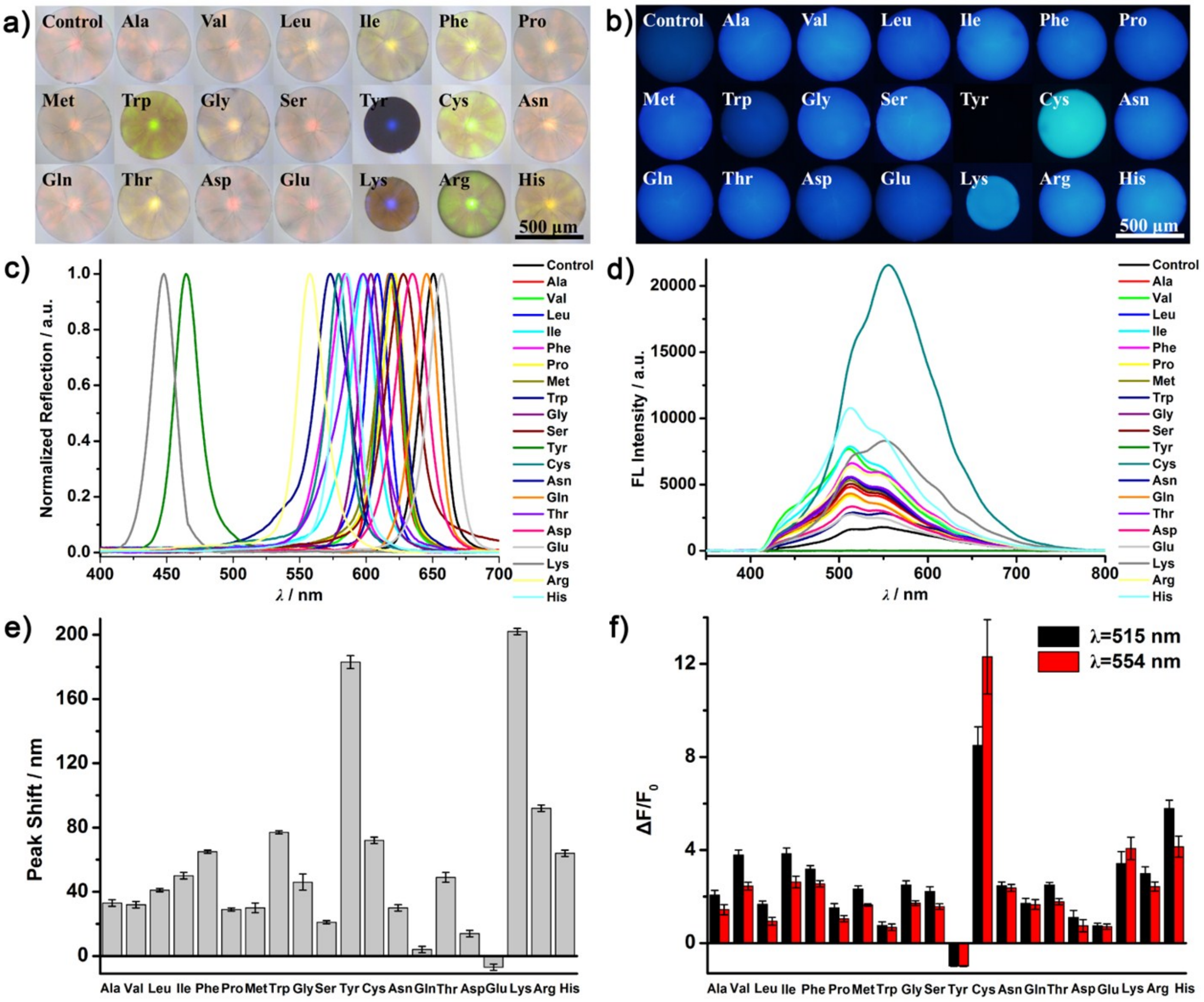

Fig. S7 PhC channel responses of AIE-doped PIL photonic spheres (OH^- form) to 20 natural amino acids at 1 mM: a) optical images, c) reflection spectra, e) histogram of reflection peak shifts; FL channel responses of AIE-doped PIL photonic spheres (OH^- form) to 20 natural amino acids at 1 mM: b) fluorescence images, d) emission spectra, f) histogram of the folds of fluorescence enhancement. The exposure time for capturing FL images is 30 ms while the integration time for recording FL spectra is 200 ms.

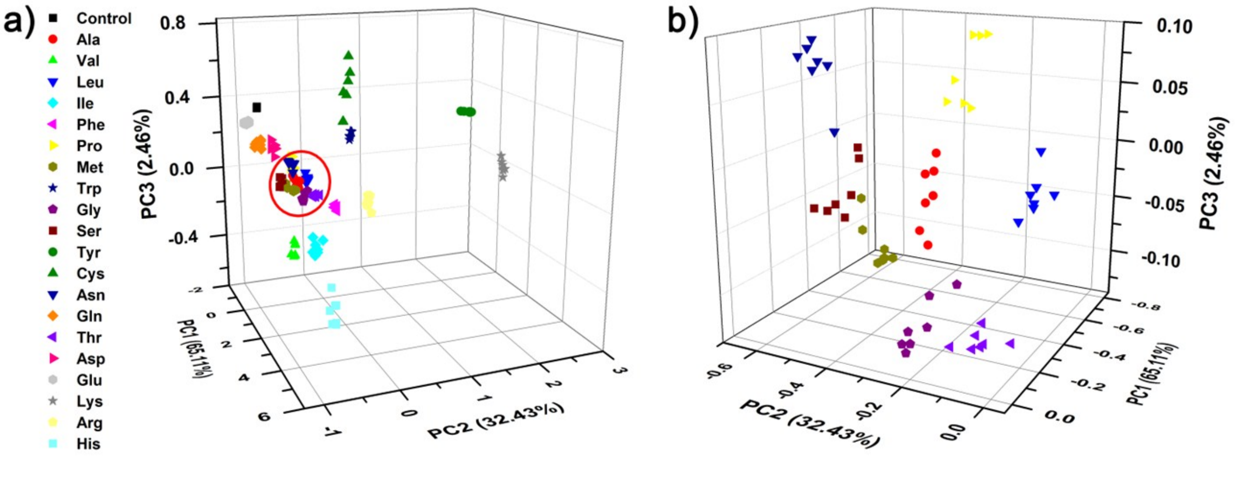

Fig. S8 AIE-doped PIL photonic spheres of OH^- form for the discrimination of 20 natural amino acids at 1 mM: a) 3D PCA plot; b) corresponding magnified plot of the red circle region in a).

Spheres of OH^- form for the discrimination of mixtures of amino acids

AIE-doped PIL photonic spheres of OH^- form were incubated in 1 mL mixtures of amino acids for three days at room temperature under continuous stirring on an orbital shaker to achieve reaction equilibrium. The concentration of individual amino acid in an arbitrary mixture was 10 mM.

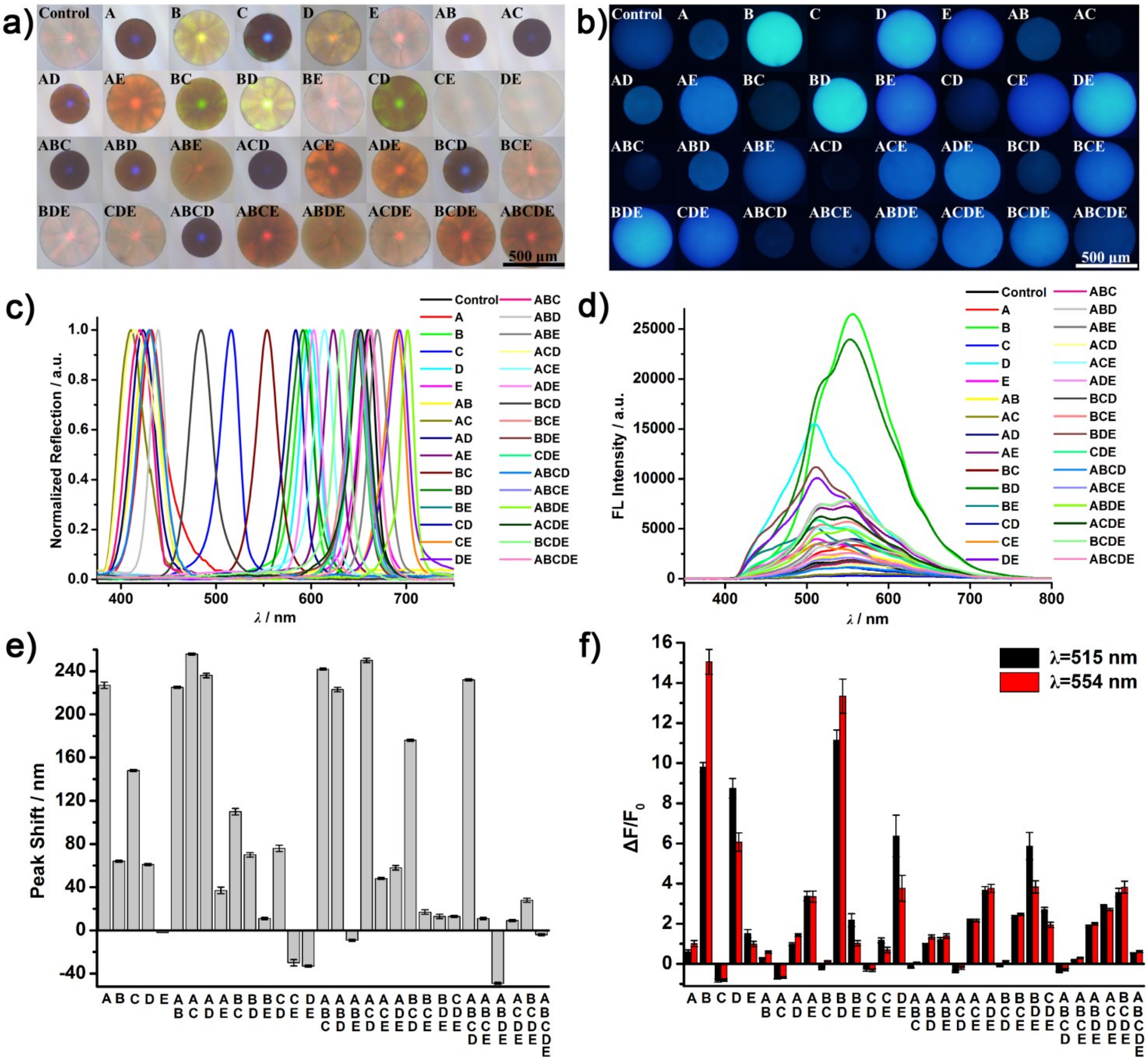

Fig. S9 PhC channel responses of AIE-doped PIL photonic spheres (OH^- form) to 1 control analyte, 5 unitary analytes and 26 mixture analytes: a) optical images, c) reflection spectra, e) histogram of reflection peak shifts; FL channel responses of AIE-doped PIL photonic spheres (OH^- form) to 1 control analyte, 5 unitary analytes and 26 mixture analytes: b) fluorescence images, d) emission spectra, f) histogram of the folds of fluorescence enhancement. A-E represent Lys, Cys, Trp, Ile and Glu, respectively. The exposure time for capturing FL images is 30 ms while the integration time for recording FL spectra is 200 ms.

Table S1 The identification of 15 blind samples which have been tested as training data sets before.

Sample#	Identification	Verification
1	AE	AE
2	BDE	BDE
3	C	C
4	ABCD	ABCD
5	Control	Control
6	BD	BD
7	ADE	ADE
8	AC	AC
9	E	E
10	ACD	ACD
11	BC	BC
12	ABE	ABE
13	DE	DE
14	ABDE	ABDE
15	D	D
100% identified		

Part 3 Spheres of OH^- form for the discrimination of phosphate derivatives

AIE-doped PIL photonic spheres of OH^- form were incubated in 1 mL phosphate derivative solutions for 6 hours at room temperature under continuous stirring on an orbital shaker to achieve reaction equilibrium. The concentration of phosphate derivatives was 10 μM.

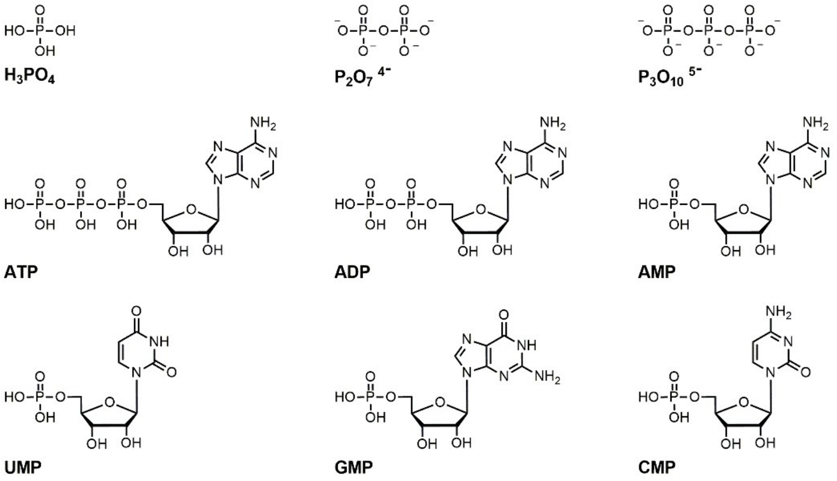

Fig. S10 Chemical structures and name abbreviations of nine phosphate derivatives.

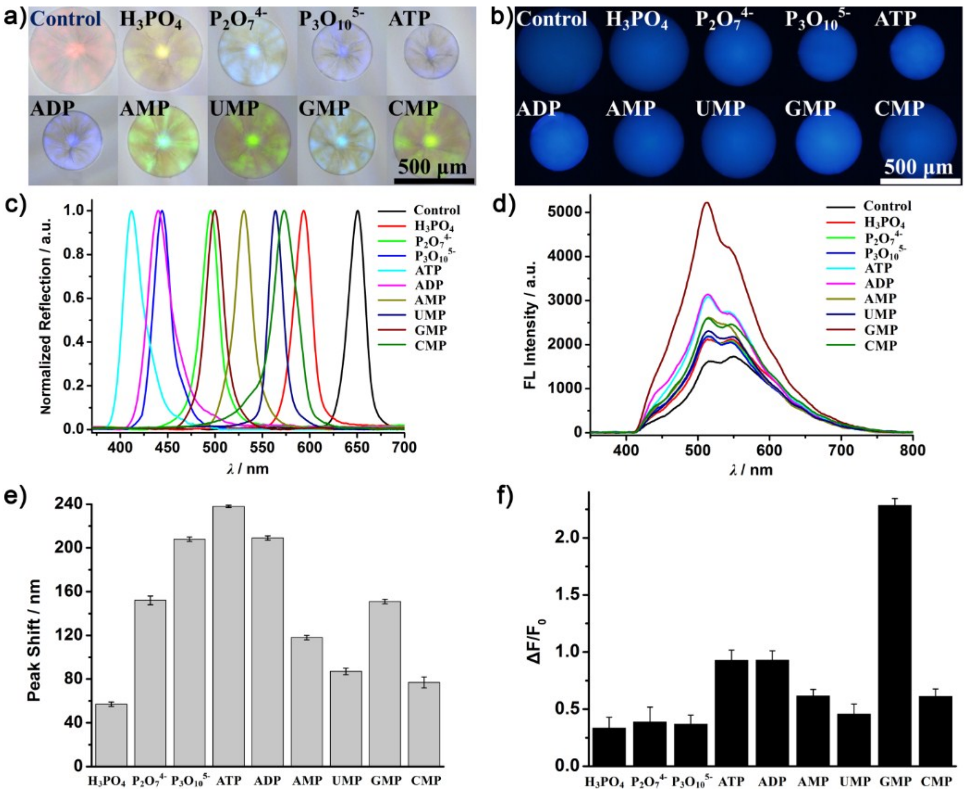

Fig. S11 PhC channel responses of AIE-doped PIL photonic spheres (OH^- form) to nine phosphate derivatives at 10 μM: a) optical images, c) reflection spectra, e) histogram of reflection peak shifts; FL channel responses of AIE-doped PIL photonic spheres (OH^- form) to nine phosphate derivatives at 10 μM: b) fluorescence images, d) emission spectra, f) histogram of the folds of fluorescence enhancement. The exposure time for capturing FL images is 30 ms while the integration time for recording FL spectra is 200 ms.

Part 4 customizable spheres of CSBA form for the discrimination of chiral dicarboxylic acids

The chiral spiral borate lithium was synthesized according to a reported method.[7] The parent AIE-doped PIL photonic spheres of Br^- form were soaked in 0.5 M chiral spiral borate lithium aqueous solution for 24 h to convert the counteranions from the form of Br^- to chiral spiral borate anion (CSBA). After this counteranion conversion, disappearance of Br peaks (11.6 wt%) at 1.340 and 1.480 keV, detection of B (8.0 wt%) at 0.183 keV, as well as typical adsorption bands of CSBA at 1720, 1494 and 694 cm^{-1} were observed, indicating the complete conversion of Br^- to CSBA.

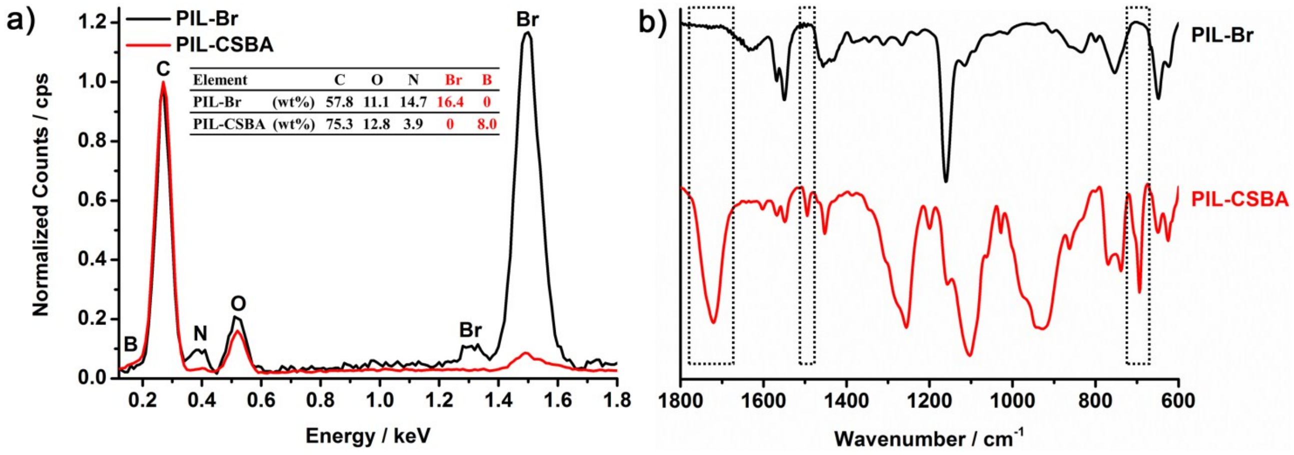

Fig. S12 a) EDX spectra of PIL-Br and PIL-CSBA; b) FTIR spectra of PIL-Br and PIL-CSBA. PIL-Br and PIL-CSBA represent PIL photonic spheres of Br^- and CSBA form.

AIE-doped PIL photonic spheres of CSBA form were incubated in 1 mL chiral dicarboxylic acid solutions for 12 hours at room temperature under continuous stirring on an orbital shaker to achieve reaction equilibrium. The concentration of chiral dicarboxylic acids was 10 mM.

Part 5 customizable spheres of citrate form for the discrimination of metal ions

The parent AIE-doped PIL photonic spheres of Br^- form were soaked in 1 M sodium citrate (CA) aqueous solution for 24 h to convert the counteranions from the form of Br^- to CA. After this counteranion conversion, 47 nm Bragg peak shift, disappearance of Br peaks (16.4 wt%) at 1.340 and 1.480 keV, typical adsorption band of CA at 1367 cm^{-1} were observed, indicating the complete conversion of Br^- to CA.

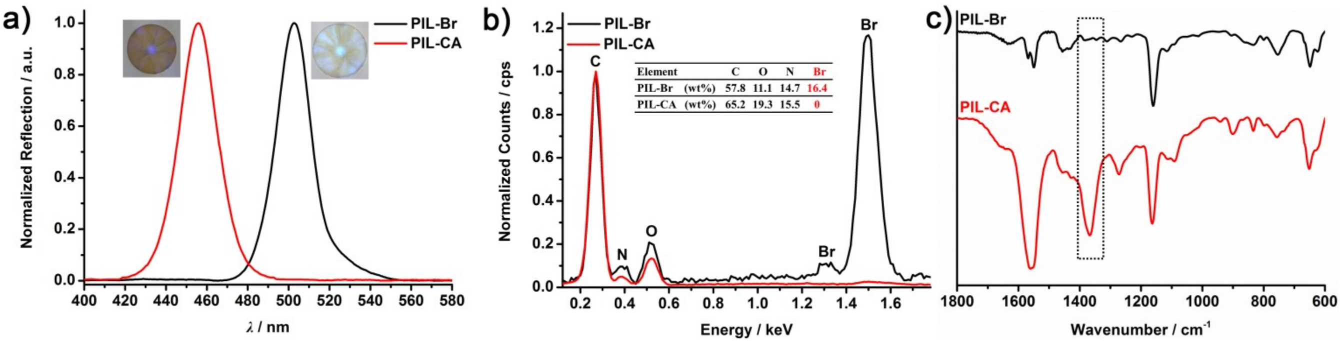

Fig. S13 a) Reflection spectra and corresponding inserted optical images of PIL-Br and PIL-CA; b) EDX spectra of PIL-Br and PIL-CA; c) FTIR spectra of PIL-Br and PIL-CA. PIL-Br and PIL-CA represent PIL photonic spheres of Br^- and CA form.

AIE-doped PIL photonic spheres of citrate form were incubated in 1 mL metal ion solutions (pH = 5, 10 mM PBS buffer) for 1 hour at room temperature under continuous stirring on an orbital shaker to achieve reaction equilibrium. The concentration of metal ions was 100 µM. All ten metal ions (Al^{3+}, Ba^{2+}, Cd^{2+}, Co^{2+}, Cu^{2+}, Hg^{2+}, Ni^{2+}, Pb^{2+}, Sn^{2+}, Zn^{2+}) were used in the form of chloride.

References

[1] T. Kakibe and H. Ohno, *J. Mater. Chem.*, 2009, **19**, 4960-4964.
[2] J. Cui, N. Gao, J. Li, C. Wang, H. Wang, M. Zhou, M. Zhang and G. Li, *J. Mater. Chem. C*, 2015, **3**, 623-631.
[3] W. Guan, W. Zhou, C. Lu and B. Z. Tang, *Angew. Chem. Int. Ed.*, 2015, **54**, 15160-15164.
[4] J. Cui, W. Zhu, N. Gao, J. Li, H. Yang, Y. Jiang, P. Seidel, B. J. Ravoo and G. Li, *Angew. Chem. Int. Ed.*, 2014, **53**, 3844-3848.
[5] (a) H. Giesche, *J. Eur. Ceram. Soc.*, 1994, **14**, 189-204; (b) H. Giesche, *J. Eur. Ceram. Soc.*, 1994, **14**, 205-214; (c) K. D. A. Hartlen, P. T. Athanasopoulos and V. Kitaev, *Langmuir*, 2008, **24**, 1714-1720.
[6] Z. Si, L. Qiu, L. Dong, F. Gu, Y. Li and F. Yan, *ACS Appl. Mater. Interfaces*, 2014, **6**, 4346-4355.
[7] S. Yu, S. Lindeman and C. D. Tran, *J. Org. Chem.*, 2008, **73**, 2576-2591.
